# Metallography of Quasicrystals in Al-Alloys

**DOI:** 10.3390/ma18194575

**Published:** 2025-10-01

**Authors:** Tonica Bončina, Franc Zupanič

**Affiliations:** Faculty of Mechanical Engineering, University of Maribor, Smetanova ulica 17, SI-2000 Maribor, Slovenia; tonica.boncina@um.si

**Keywords:** metallography, quasicrystal, aluminium, microstructure, microscopy

## Abstract

Quasicrystals are ordered phases without periodicity. They are often found in aluminium and other alloys. They can appear in different sizes. Therefore, several metallographic and characterisation techniques are required to fully determine their shape, size, crystallography, and chemical composition. This review paper gives special attention to identifying quasicrystals in aluminium alloys using classical metallographic techniques, such as etching, deep etching, and particle extraction, which allow the investigation of larger areas by light and scanning electron microscope, giving additional information by combining with complementary high-resolution techniques.

## 1. Introduction

It has been 40 years since Shechtman et al. [1] published the paper “Metallic phase with long-range orientational order and no translational symmetry” after discovering a new phase in Al-Mn melt-spun ribbons. Later, this fascinating metallic phase obtained the name “quasicrystal” [2], which is widely used. Electron diffraction in TEM was used for determining its structure [3,4]. Therefore, TEM and its complementary techniques (EDS and EELS) still play a vital role in quasicrystal characterisation. The quasicrystals embedded in a metallic matrix can have sizes as large as a few millimetres, so they could be resolved by the naked eye or as small as a few nanometres, requiring high-resolution microscopy techniques [5]. The wide range of sizes requires other characterisation techniques besides TEM.

### 1.1. The Significance of Metallography

Metallography is a scientific discipline that deals with the metallographic preparation of samples, their analysis, and the identification of microstructural constituents in metals, alloys, and other materials [6]. Classical metallographic preparation and microscopy of the metallic sample have remained the primary methods for characterising and developing new metallic materials and for the quality control of materials in the metallurgical and metal processing industries [7].

A landmark year for metallography was 1863, when Henry Clifton Sorby succeeded in preparing a metallic sample by grinding, polishing, and etching. It was suitable for the analysis with a light microscope. He stated that he opened a new field of research, namely “microscopic metallurgy”. He wrote in his diary that he had discovered the structure of steel. Later, the method was developed for all metals, including aluminium, and is still the primary method for determining 2D microstructures. Metallographic research has played an essential role in industrial development, then and now [8]. Probably the first comprehensive review of the metallography of aluminium alloys was prepared by Anderson in 1919 [9].

Several research methods have been applied to metallography. Exner systematically described a list of metallographic methods [10]. He divided the methods into metallographic sample preparation, optical, quantitative, chemical, surface and dynamic metallographic methods, and visual metallography. A significant milestone in metallography was the invention of scanning electron microscopes, which allowed for better resolution, higher magnification, and an extraordinary depth of field compared to light microscopy [11]. However, metallographic preparation for scanning electron microscopy has not changed a lot. The grinding, polishing and etching methods are still widely used in scientific research and industrial materials control [12].

Advances in electron microscopy, with the introduction of field-emission scanning microscopes with a resolution of less than 0.5 nm, have brought a broader awareness of the adverse effects of rough grinding and aggressive etching on the original material’s microstructure. This notion indicated the necessity for changing established sample preparation methods and analytical procedures. The latter is significant when revealing and identifying nanoparticles by SEM [13].

### 1.2. Quasicrystal Structure

Quasicrystal is a unique state of matter exhibiting an ordered structure without periodicity. Quasicrystals were discovered in the 1980s [1]. Their finding resulted in a considerable paradigm shift regarding the structure of the solids [14]. Mathematicians discovered aperiodic tiling in the early 1960s [15], but nobody believed they would appear in the physical world. Sharma [16] briefly described the history of quasicrystal research. Even after their discovery, Linus Pauling, a famous scientist and a two-time Nobel winner, tried to disprove their existence [17].

**Figure 1:**
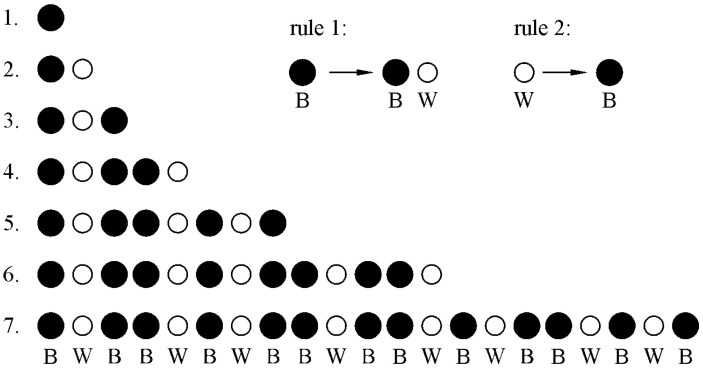
Quasiperiodicity is shown as a sequence of black and white circles (Fibonacci chain). The distribution in the next generation is the sum of the two previous generations. The sequence has a long-range order without periodicity [18].

For most non-mathematicians, the basic characteristics of quasicrystals are hard to understand when dealing with them for the first time. They can be easily comprehended by making sequences of black (B) and white (W) circles using two simple rules (Figure 1). We start with just one black circle in the first generation. The sequence in the next generation can be obtained by changing one black circle to a black and white circle (rule 1) and a white circle to a black circle (rule 2). We can see that the sequence is not periodic, but the colour of a circle at any position can be unambiguously predicted. The sequence has a long-range order without periodicity. The sequence *S* at generation *n* is the sum of the sequences of the two previous generations:

*S_n_* = *S_n_*_−2_ + *S_n_*_−1_ (1)

The number of circles in each generation follows the Fibonacci sequence: 1, 2, 3, 5, 8, 13, 21, 34…

**Figure 2:**
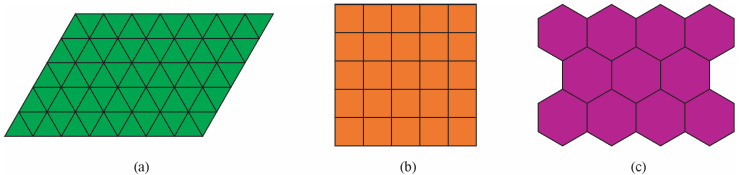
Periodic tiling (**a**) with triangles (threefold rotational symmetry), (**b**) squares (fourfold rotational symmetry), and (**c**) hexagons (sixfold rotational symmetry) [18].

Periodic crystals have a repeating unit cell extending in all three spatial dimensions, creating a long-range order. They have two-, three-, four-, or sixfold rotational symmetries. Tiles with these symmetries can completely cover the surface (Figure 2). They have sharp diffraction patterns with the same symmetries. On the contrary, amorphous materials lack any long-range order. However, they have a short-range order. Their diffraction patterns are typically diffuse, without sharp peaks.

**Figure 3:**
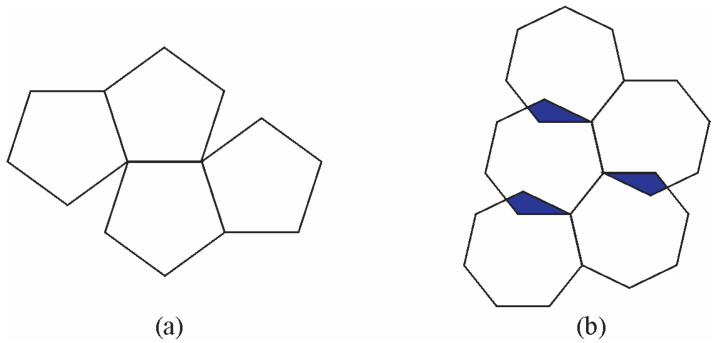
Aperiodic tiling with (**a**) pentagons (they fail to cover the whole area) and (**b**) septagons (they overlap) [18].

Quasicrystals have non-crystallographic symmetries. Tiles with such symmetries cannot cover the whole surface or overlap (Figure 3).

**Figure 4:**
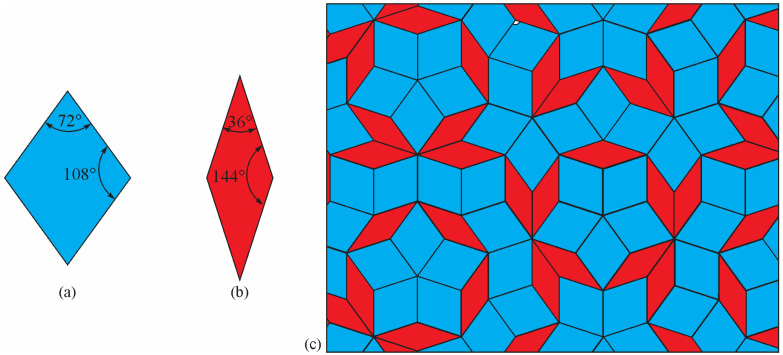
Tiling by two types of rhombi using (**a**) a thick rhombus and (**b**) a thin rhombus. (**c**) Penrose quasiperiodic tiling with local fivefold symmetries was obtained using thick and thin rhombi, as shown in (**a**,**b**), with special matching rules [18].

Since non-crystallographic shapes cannot cover a surface completely, Penrose [19] used two types of rhombi to achieve full coverage (Figure 4). He obtained a 2D quasiperiodic structure by applying special matching rules.

The Penrose 2D quasicrystal tiling displays a non-repeating, aperiodic pattern that can fill space without gaps. It also has a sharp diffraction pattern but non-crystallographic or forbidden symmetries. Experimentally, quasicrystals with five-, eight-, ten-, or twelvefold symmetries were discovered [20,21,22,23]. Mackay [24] experimentally proved that Penrose tiling has a sharp diffraction pattern and provided a formalism for its description.

The real quasicrystals consist of aperiodic arrangements of clusters. On the other hand, quasicrystalline approximants (QAs) consist of the periodic arrangement of the same clusters, enabling crystallographers to identify their structure. The usual classification of quasicrystal structures is based on the prevalent cluster type in the structure [14]. This can be the Mackay or pseudo-Mackay cluster (type A) [25,26,27], the Bergman cluster (type B or Frank–Kasper type) [28,29], and the Tsai (type C) [30] cluster. However, icosahedral quasicrystals of type A cannot be adequately described based on Mackay clusters alone. They can be slightly better described with Bergman clusters (type B). When only one of these two cluster types is used, only ≈75% of the structure is covered, but it is increased to ≈98% when both types are used simultaneously. The remainder represents glue atoms [31].

Real quasicrystals also have different defects, such as phasons [32,33,34,35], dislocations [36,37,38], vacancies [39,40], antiphase boundaries [41], stacking faults [42,43], cracks [44,45,46], and growth defects [46]. Defects affect quasicrystals’ physical, chemical, and mechanical properties, like in periodic crystals. Each defect type has its specific effect on properties. We can mention phason defects, which are specific defects in quasicrystals, arising from rearrangements in quasiperiodic atomic order. They, for example, reduce electric conductivity and affect plastic deformation and magnetic properties [47].

**Figure 5:**
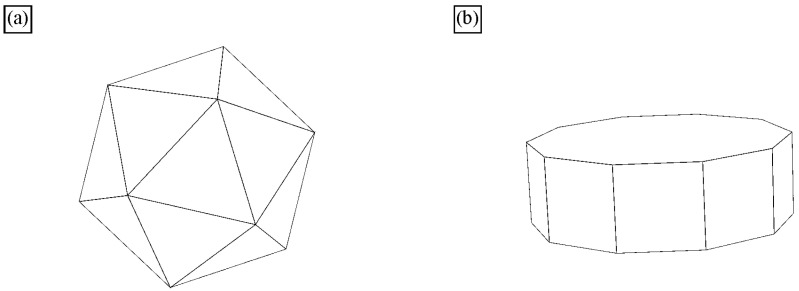
Basic symmetry units of IQC and DQC. (**a**) An icosahedron and (**b**) a decagonal prism [18].

All quasicrystals were made artificially, while natural quasicrystals were discovered later [48,49]. The quasicrystals in the Al-Mn system are metastable and transform into stable phases during annealing [50]. On the other side, many systems have stable quasicrystals, including Al-Cu-Fe [51]. Thus, the quasicrystalline phase appears in the appropriate phase diagram [50]. Stable quasicrystals can be grown from the melt. Such single quasicrystals are typically faceted and possess shape characteristics for five-, eight-, ten-, and fivefold symmetries. The most typical shapes are icosahedra, pentagonal dodecahedra, and decagonal prisms (Figure 5). Their sizes are between mm and cm. They can be observed by the naked eye or with a magnifying glass. They are used to determine their electronic, mechanical, physical, and other properties. The examples of some quasicrystals are given in Table 1. The quasicrystals normally form in binary and multicomponent alloys. Single-element quasicrystals can form under special conditions. For example, plasmonic silver nanoparticles can have a shape of icosahedron [52], silicon quasicrystal can form on glass [53], while quasicrystalline monolayers of Sb and Bi form on the icosahedral Al-Pd-Mn and decagonal Al-Ni-Co surfaces [54].

There are two large types of quasicrystals: 2D and 3D quasicrystals [14]. The former has three groups, i.e., eightfold octahedral, tenfold decagonal, and twelvefold dodecagonal. However, only the fivefold icosahedral type is found as the latter. Icosahedral quasicrystals are quasiperiodic in all directions. Their structure is based on an icosahedron, a body with twenty faces, all triangles (Figure 5a). An icosahedron can be regarded as a projected polyhedron of a 6D cube into the 3D space. It has 12 vertices with fivefold symmetry, 20 triangular facets with threefold symmetry and 30 edges with twofold symmetry. Since the icosahedron is centrally symmetrical, the number of independent symmetric axes is half of the above numbers, i.e., 6 for fivefold, 10 for threefold, and 15 for twofold. This makes the icosahedron a body with a very high degree of symmetry. Decagonal quasicrystals (DQCs) are 2D quasicrystals. They are quasiperiodic in one plane, thus in two directions, but periodic in the third direction. The basic unit is a decagonal prism (Figure 5b). The decagonal prism has a tenfold axis perpendicular to the basal plane and ten twofold axes. A set of fivefold axes passes through the centres of the opposite vertical facets, while a set of other fivefold axes goes through the centres of opposite vertical edges [56].

**Figure 6:**
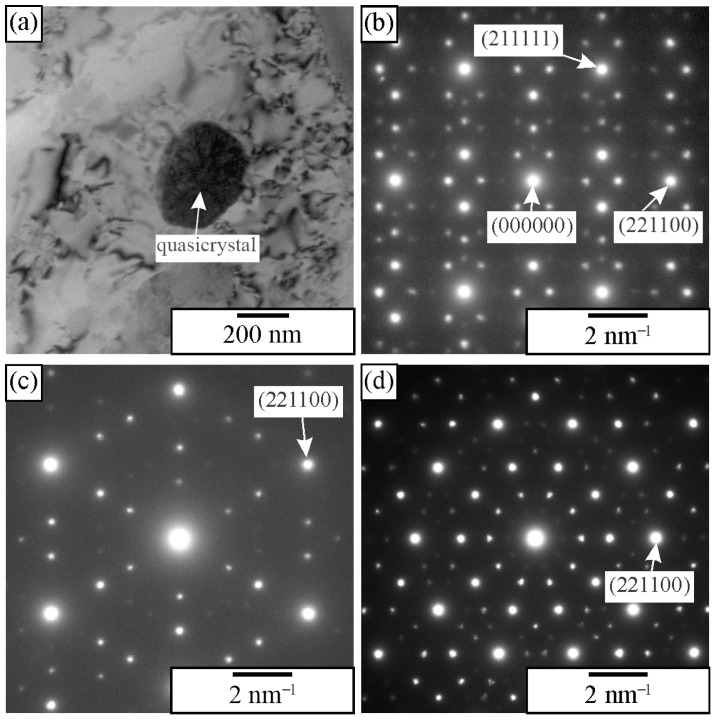
Identification of IQC using electron diffraction in TEM [59]. (**a**) A quasicrystalline particle in α-Al (Al-rich solid solution). (**b**) Twofold, (**c**) threefold, and (**d**) fivefold diffraction patterns. The most prominent spots are indicated.

The best way to identify quasicrystals is through electron diffraction in TEM (Figure 6). The diffraction patterns of an IQC particle with the zone axes parallel to two-, three-, and fivefold axes correspond to Figure 6b–d, respectively. A system with six indices is used to index icosahedral quasicrystals. For details, a reader should consult, for example, references [14,57,58]. In order to be able to index the reciprocal lattice spots as a combination of integers, at least six basis vectors are required for the icosahedral phase, and five for the decagonal phase. Elser [57] selected the six vectors for indexing the IQC diffraction pattern, which can be obtained by a projection of a cube in six dimensions to three dimensions. The Bragg vector for each diffraction peak is expressed as a linear combination of basis vectors multiplied by integer indices, scaled by a factor called the quasilattice parameter. The observable reciprocal space spot nearest to the transmitted beam along a fivefold direction (vertex vector) is indexed as (100,000).

**Figure 7:**
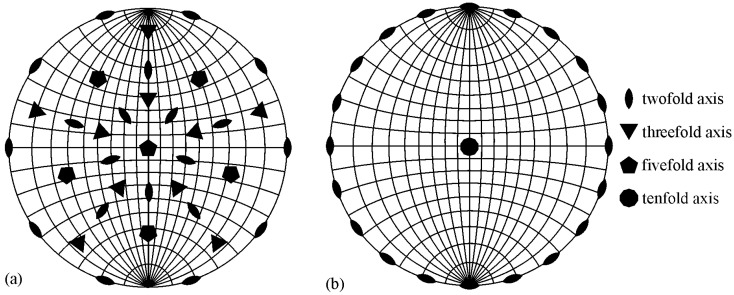
Stereographic projection of (**a**) the icosahedral space group along a fivefold axis and (**b**) the decagonal space group along the tenfold axis [18].

Figure 7 shows that these axes also appear in its stereographic projection. The periodic crystals also have two- and threefold axes; hence, their diffraction pattern is periodic. However, quasicrystals do not have periodic diffraction patterns. The distances to the main peaks typically increase with the golden mean *τ*, which is the ratio between a diagonal and an edge of a pentagon:

(2) $$\tau =\frac{1+\sqrt{5}}{2}$$

Twofold diffraction patterns also allow the calculation of a quasicrystalline constant [57,58].

**Figure 8:**
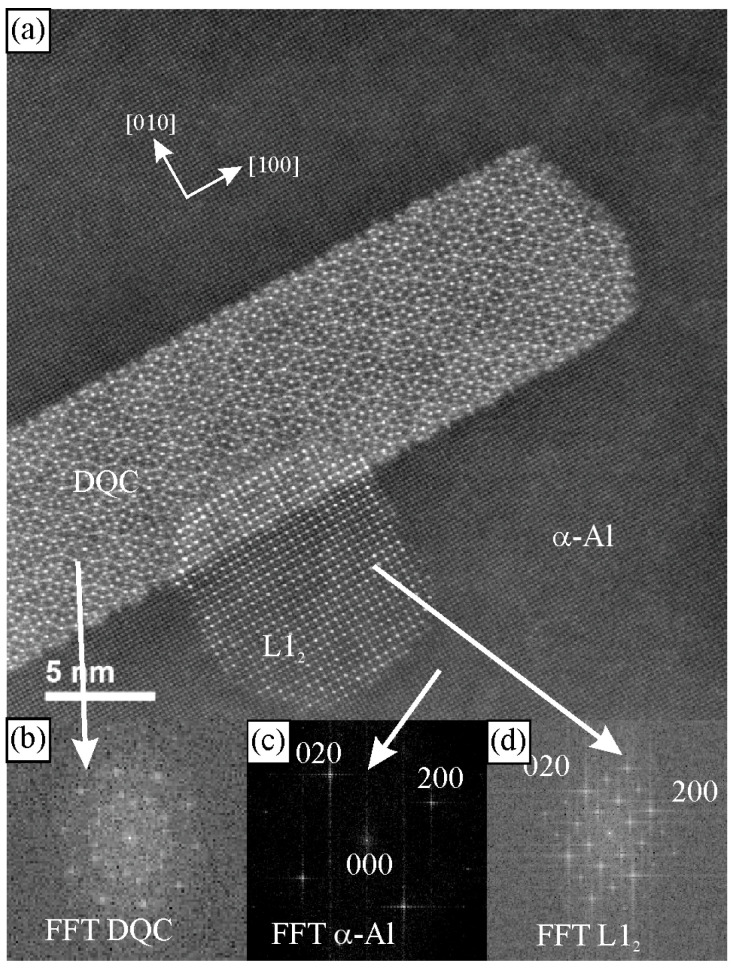
Identification of DQC in a multiphase microstructure using electron diffraction in TEM. (**a**) A STEM high-resolution HAADF micrograph, (**b**) DQC tenfold FFT, (**c**) α-Al matrix fourfold FFT, and (**d**) L1_2_ precipitate fourfold FFT [60].

Figure 8 shows the image, the diffraction pattern of DQC in a periodic α-Al matrix, and the presence of the ordered L1_2_ structure. The quasiperiodic structure is evident (Figure 8a), and the diffraction pattern (Figure 8b) shows the tenfold rotational symmetry.

### 1.3. Stability of Quasicrystals

The quasicrystal can be thermodynamically stable or metastable. Here are some examples of stable quasicrystals: Al-Cu-Fe [61,62], Al-Mn-Pd, Al-Cu-(Co-Rh), Ag-In-Ca [63], Al-Ni-Co [64], Cd-Mg-Dy [65], Cd-Ca [30], Al-Ni-Rh [66], Al-Cu-Co [67], Al-Co-Ni [68], Al-Pd-RE (RE—rare-earth metal) [69], and metastable ones, Al-Mn [70], Al-Fe [71], Al-Cr [72,73], Al-V [74], and Al-Ni [75]. The readers can obtain much more detailed information about stability and properties of quasicrystals in “Comprehensive experimental datasets of quasicrystals and their approximants” [55].

Quasicrystals are thermodynamically stable when their free energy is lower than that of competing phases. This is achieved through contributions from harmonic and anharmonic vibrations, chemical substitution disorder, and electronic excitations [76]. Thermodynamic stability of quasicrystals often depends on the electron per atom ratio (e/a), typically around 1.7 to 1.8 for stable phases [61,77], the atomic size factor [61,76], and entropy contribution. In the Al-Mn system, the entropy contribution due to the presence of icosahedral short-range order in the liquid phase can facilitate the formation of quasicrystals upon cooling. Generally, it prevents the transition of quasicrystals to more stable crystalline phases [78,79]. The work of Mihalkovic and Widom [76] suggests that the Al-Cu-Fe quasicrystal can be a truly stable phase even at 0 K; other stable quasicrystals can be stable at least at higher temperatures due to entropy stabilisation. The stability of a quasicrystal can be influenced by alloying. So et al. [69] studied the (Al, Ga)-Pd-RE systems, using the rare-earth elements Yb, Tm, and Er. They found that a stable IQC phase is at 2.10 valence electrons per atom, and that the stability of IQC phases becomes lower with increasing atomic radii of the RE metals.

According to stability, quasicrystals can be formed by different manufacturing processes. The most typical are rapid solidification [62,80,81], mechanical alloying [82,83,84,85], powder metallurgy and sintering [86,87,88,89,90,91], arc melting [92], additive manufacturing [93,94,95,96], precipitation hardening [97,98], thermal spray coating [99,100,101,102], and casting [103,104,105,106,107].

### 1.4. Properties and Applications of Quasicrystals

Quasicrystals have a long-range quasiperiodicity and, at the same time, a complex local atomic order independent of their long-range structure. Thus, there is often a controversy about which effect is most important for the properties of quasicrystals [108].

In the electronic structure of quasicrystals, there is a pseudogap at the Fermi level, indicating that the density of electronic states is zero. The presence of a pseudogap is a key factor influencing the electron transport. It strongly reduces electrical conductivity; thus, electron transport resembles semiconductor-like transport, not metallic conductivity [109].

The pseudogap also determines the magnetic behaviour of quasicrystals. Some quasicrystals exhibit temperature-dependent susceptibility consistent with pseudogap-induced electronic states near the Fermi level [110,111,112,113]. In magnetic quasicrystals, the spin-glass effect is often observed [114]. It arises in systems with competing interactions—for example, when some neighbouring spins prefer ferromagnetic alignment and others prefer antiferromagnetic alignment, leading to frustration. The system cannot settle into a single ground state but becomes trapped in many nearly degenerate configurations [114].

Thermal conductivity exhibits unusually low values with pronounced anisotropy linked to stacking periodicity and cluster-mode phonon scattering [115,116].

Quasicrystals also have a very low friction coefficient, often lower than conventional metals or ceramics, making them promising for wear-resistant coatings and tribological applications [117]. The unusual friction behaviour arises from their electronic structure (pseudogap reduces their metallic nature and adhesion), atomic surface arrangement, lack of translational periodicity (they contain phasons, which can accommodate atomic rearrangements under stress), high hardness, and formation of thin oxide films on the surface [118].

QCs can also be used for thermoelectric applications. Thermoelectric behaviour is explained by their cluster hierarchy and covalent chemical bonding in clusters [119,120].

The quasiperiodic lattice and cluster structures lead to high hardness, elastic moduli, and complex deformation behaviours [121]. Elastic and plastic deformation are influenced by phason strain fields and dislocation glide mechanisms [122]. Dislocations also cause plastic deformation of single quasicrystals. However, quasicrystals show their brittle character at low temperatures, but at higher temperatures, their ductility increases [123,124]. Nanostructured quasicrystalline coatings exhibit enhanced mechanical performance and damping capacities [125]. Observations of inverse Hall–Petch behaviour in nanophase quasicrystals highlight novel grain size effects on mechanical stability [126]. In situ formed and ex situ added quasicrystals can considerably strengthen the metallic [127,128,129] and polymeric [130] matrices. In Al-alloys strengthened by quasicrystals, adding rare-earth elements considerably enhances mechanical properties. Ce [101,127,128] and mischmetal [129] were the most investigated additions. Mechanical strength exceeding 1000 MPa was attributed to grain refinement, precipitation hardening, and load transfer from nanoscale quasicrystals, although ductility decreases at higher rare-earth contents [131].

Possible applications of quasicrystals are presented in many references [117,132,133,134,135]. Yet, challenges remain in synthesising defect-free, large-scale QCs for industrial use [117]. Table 2 gives some of the most important properties of quasicrystalline alloys.

### 1.5. The Aim of This Review

Quasicrystals are rarely present as a single-phase material. They are usually embedded in the matrices of different metals. They can appear in various sizes, including the nanoscale and macroscale regions. Analytical TEM is primarily used for its characterisation since it allows the detailed determination of quasicrystals’ crystallographic, chemical, and geometrical characteristics. However, TEM samples are very small and embrace only a small part of a specimen, e.g., a cast part. Therefore, it is also convenient to use other methods to help identify quasicrystals and their distribution in much larger areas than those investigated by TEM. This review paper gives special attention to identifying quasicrystals in aluminium alloys using classical metallographic techniques, such as etching, deep etching, and particle extraction, which allow the investigation of larger areas by light and scanning electron microscope, giving additional information by combining with complementary high-resolution techniques. The first part deals with the preparation of samples for light microscopy and scanning electron microscopy, as well as identifying quasicrystals from 2D cross-sections. Special attention is given to identifying quasicrystals formed during solidification and precipitation from the solid solution. The second part is devoted to direct 3D characterisation of quasicrystals using deep etching and particle extraction methods. The aim is also to give information regarding alternative techniques.

## 2. Light and Scanning Electron Microscopy of Polished and Lightly Etched Samples

### 2.1. Classical Metallographic Preparation of Aluminium Alloys

Classical metallographic preparation consists of grinding, polishing, and etching [143]. The main goal of the preparation steps is to reveal the sample’s actual microstructure. It has been a principal method for characterising and developing new metallic materials. Metallographic preparation is also widely applied for the quality control of materials in the metallurgical and metal processing industries [144]. Chemical etching is a controlled process that selectively removes material from a solid surface by chemical reactions, often using acids, bases, or reactive gases [143]. When applied at the micro- and nanoscale, chemical etching can alter the surface’s topography, roughness, and chemical composition. Light microscopy is mainly used to observe microstructures, which normally requires relatively strong etching to reveal microstructural constituents [143,145]. Due to strong etchants, some microstructural constituents are likely attacked too much, but this cannot be visible in a light microscope due to rather low magnifications (up to 2500×). Some etchants can dissolve Mg_2_Si in Al-Mg-Si alloys [146], while others can dissolve iron-rich phases [147], leaving holes on the surface, interpreted as black-coloured phases. The advent of field-emission scanning microscopes with a resolution of less than 1 nm and the application of excellent backscattered electron detectors have considerably reduced the need for etching [148].

Most etchants for metals were developed by the 1980s and are described in the following books:

- George, F., Vander, Voort. *Metallography Principles and Practice* [143] and
- ASM Handbook, Volume 9, *Metallography and Microstructures* [149].

They are used in many studies on quasicrystalline alloys. The most used etchants are given in Table 3.

The metallographic preparation of aluminium alloys usually follows general procedures. It often starts with sawing the samples to appropriate sizes, hot or cold mounting into a polymeric resin, grinding with SiC papers, and polishing with different diamond pastes or alumina suspensions (usually 9, 6, and 3 µm), a colloidal suspension of 0.06 µm SiO_2,_ and etching [144]. By mechanically preparing aluminium samples, one should avoid grinding with the finest grinding papers and diamond paste, since SiC and diamond particles can be incorporated into a soft aluminium matrix. The deformation layer is progressively thinned with sequential grinding and polishing steps [143]. Vibrational polishing can effectively remove the deformation layer, making samples appropriate for HR SEM, EDS, and EBSD investigations [156]. In our time, ions can also remove the deformation layer (e.g., argon ions in cross-section polishers). However, parameters should be carefully optimised to avoid the appearance of ion-induced artefacts [157]. Nevertheless, chemical etching is commonly used for light microscopy observations because the microstructure details are hardly visible without etching [158]. On the contrary, polishing without etching can give convenient results when using scanning electron microscopy [94,159]. Examining a polished surface before etching is crucial since a general overview of the microstructural constituents can be obtained. Slight etching can remove a surface deformation layer. A shallow groove formed at grain boundaries and a thin step formed at the particle/matrix interface can increase contrast and make identifying microstructural constituents easier when using a secondary electron detector. Additionally, polished surfaces allow for the most reliable microchemical analysis [160,161]. A classical strong etchant is not always appropriate for scanning electron microscopy, since it can dissolve some phases and deteriorate microstructural and chemical analyses. Thus, modification of established procedures is required [162]. Sometimes, metallographic preparation is taken for granted, and the preparation details are not given. Nevertheless, the inconvenient use of etchants can be identified by inspecting micrographs (look at SEMs in Ref. [163]). Thus, comparing polished and etched specimens is advisable to reveal potential etching artefacts. Etching is unnecessary when using a backscattered electron detector, and the phases have different backscattering coefficients (having elements with different atomic numbers Z) [148]. Thus, microstructural constituents of varying chemical compositions can be distinguished with slight etching or even without it.

This section deals with light and scanning electron microscopy of polished and lightly etched samples of aluminium alloys containing quasicrystals. This is, in fact, 2D metallography. Light microscopy can identify quasicrystals and their geometrical features when quasicrystalline particles are sufficiently large, e.g., above 5 μm. On the other hand, scanning electron microscopy can identify nanoscale quasicrystalline particles (even as small as 20 nm), with additional information obtained by EDS, WDS, or EBSD.

The basis for identifying quasicrystalline particles in a 2D section is based on the fact that the experimentally observed quasicrystals have five-, eight-, ten-, and twelvefold rotational symmetries, which are different from those of periodic crystals [164]. The shape of a crystal, as well as a quasicrystal, depends on its point group symmetry. Several shapes are possible in any point group symmetry, depending on the facets’ surface energy, growth conditions, and presence of surface-active elements. The most often observed shapes of icosahedral quasicrystals are the shapes of a pentagonal dodecahedron [165], icosahedron [166], and rhombohedral triacontahedron [167]. In this way, a well-faceted quasicrystal and non-faceted quasicrystalline dendrites can be unambiguously identified by their shape and “non-crystallographic” symmetry.

### 2.2. Quasicrystal in Melt-Spun Ribbons

**Figure 9:**
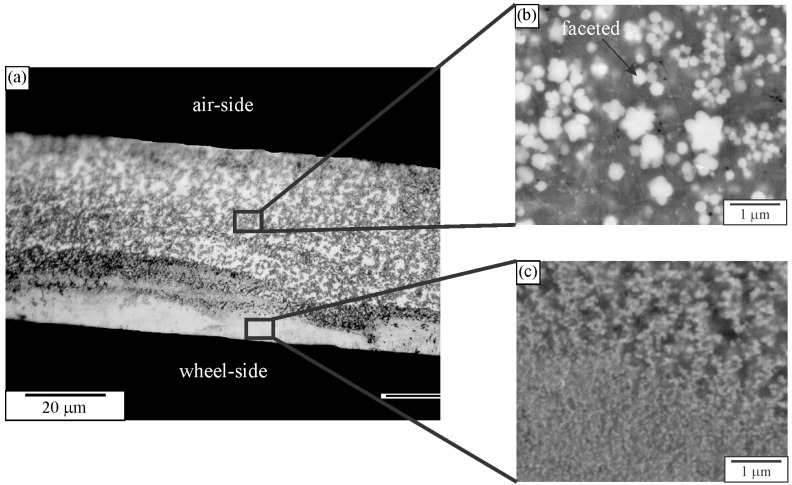
Microstructure of a melt-spun Al-Mn-Be alloy; (**a**) light micrograph and (**b**,**c**) backscattered electron micrographs (SEMs) [172].

Quasicrystals were first identified in melt-spun ribbons [1]. The microstructure typically consists of quasicrystalline particles embedded in an Al-rich solid solution, denoted by α-Al. The cooling rate by melt spinning can attain 10^4^–10^6^ K/s, enabling copious nucleation of tiny quasicrystalline precipitates [168]. They are too small to be resolved by light microscopy (Figure 9a). Even high-resolution SEM hardly resolves particles formed next to the wheel side (Figure 9c). The faceting tendency can be seen in particles as small as 300 nm. However, it is impossible to determine their exact shape according to their 2D cross-sections (Figure 9b). Thangaraj et al. [166] tried to distinguish quasicrystalline shapes (triacontahedron, icosahedron, and pentagonal dodecahedron) by imaging quasicrystalline particles in TEM along two-, three-, and fivefold axes. They found the shapes indistinguishable when projected along higher symmetry axes, like threefold and fivefold axes. However, the projection along the twofold axes enabled distinction between different shapes. Ishimasa and Nissen [169] found out by the same method that the faceted quasicrystalline particles had a form of pentagonal dodecahedron. The quasicrystals can also have a dendritic shape [169,170,171]. The determination of quasicrystal shapes in melt-spun ribbons by deep etching is given in Section 3, Deep Etching and Particle Extraction.

### 2.3. Quasicrystals in As-Cast Samples

Quasicrystals in Al-Mn alloys can form at very high cooling rates (10^5^ K/s). Casting into metallic moulds cannot produce such cooling rates; the highest cooling rates can be a few thousand K/s. Under such conditions, quasicrystals do not form, but the orthorhombic Al_6_Mn phase does [173]. Adding several alloying elements can reduce the critical cooling rate for quasicrystal formation. Kim et al. [174] obtained a quasicrystal phase in a bulk Al_93_Fe_3_Cr_2_Ti_2_ alloy. The quasicrystalline phase also appeared in the Al-Mn-Be alloy [163,175,176,177], Al-Mn-Fe-Si-V [178], and Al-Mn-Ce alloy [179]. Stan-Głowińska [180] discovered quasicrystals in Al-Mn base alloys alloyed with Cr, Co, Ni, and Cu additions. Leskovar et al. [181] created primary metastable quasicrystals in Al-Mn-Si alloys alloyed with Zn, Ca, and Sr. Primary quasicrystalline phases are also formed in magnesium alloys [182]. Thus, quasicrystals appeared in different sizes and shapes, which can be identified using metallography, as explained in detail in Ref. [183]. The primary quasicrystals can have polygonal or dendritic shapes [167,169,171,184,185]. The same or similar shapes can also appear in the other alloys.

**Figure 10:**
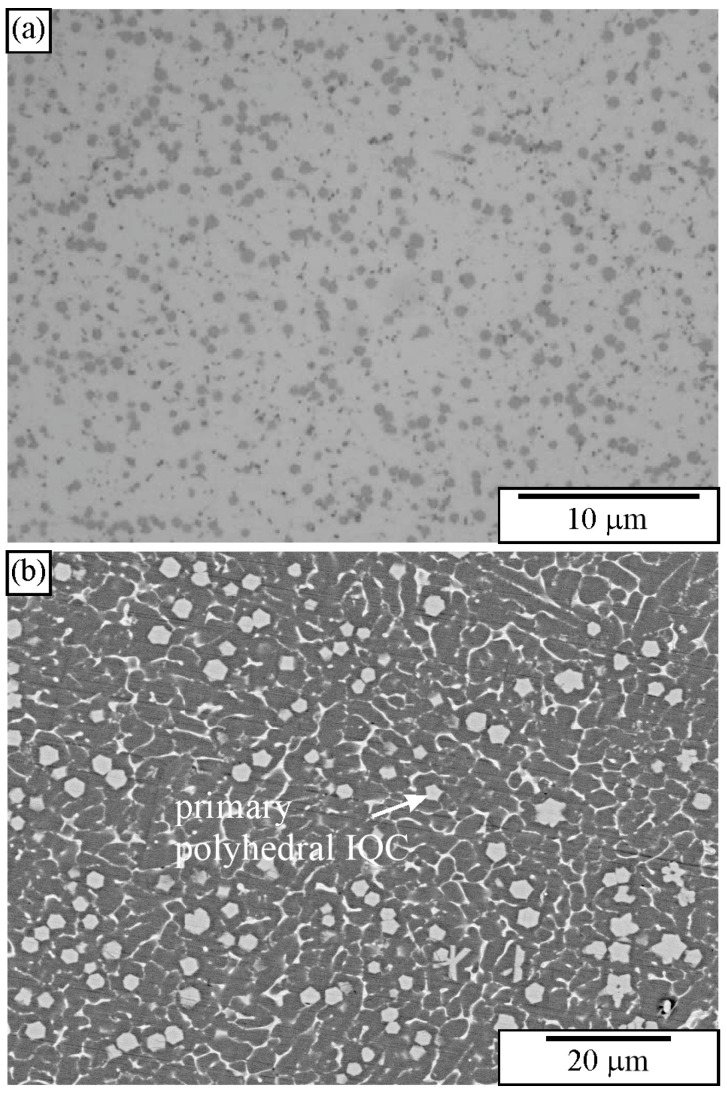
Microstructure of an Al-Mn-Be alloy after casting into a copper mould; (**a**) light micrograph and (**b**) backscattered scanning electron micrograph. Stress unetched [186].

Figure 10 shows the microstructure of an Al-Cu-Mn-Be alloy in the as-cast state. The sample was investigated in a polished condition, without any etching. The sizes of polygonal quasicrystalline particles are 2–5 μm. The light micrograph (Figure 10a) does not have sufficient resolution to reveal the characteristics of the quasicrystalline particles. On the other hand, the backscattered electron micrograph reveals some features of quasicrystalline particles (Figure 10b). Some particles are pentagonal, whereas most of them are hexagonal.

**Figure 11:**
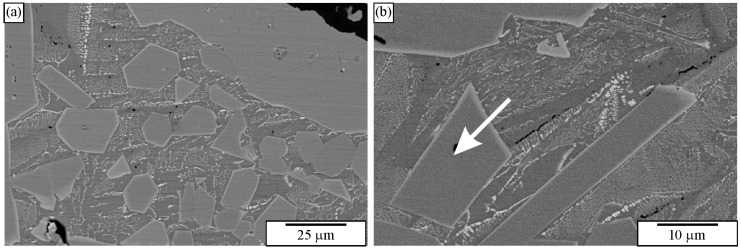
Microstructure of the alloy Al-Cu-Fe with a thermodynamically stable icosahedral quasicrystalline phase. (**a**) The area with several intersected particles; (**b**) a particle with a trapezoidal shape, indicated by the arrow, is possible only if the quasicrystalline particle has the shape of a pentagonal dodecahedron. The equilibrium shape of the quasicrystal in this alloy is the pentagonal dodecahedron [183].

Figure 11 shows a microstructure of the Al-Cu-Fe alloy with several intersecting quasicrystalline particles. The particles are much larger than the previous ones, so their shapes can be seen. The presence of the icosahedral quasicrystalline phase was determined using other methods, such as XRD and TEM [183,187].

**Figure 12:**
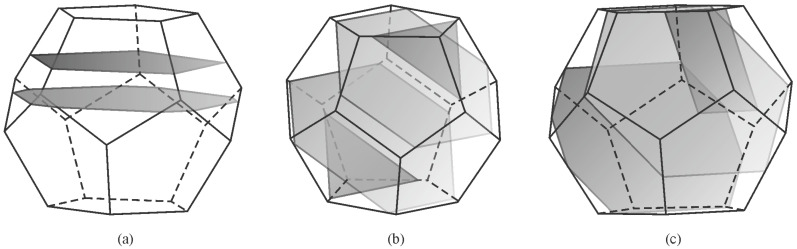
Intersections through a pentagonal dodecahedron by intersecting planes whose normal is perpendicular to (**a**) fivefold, (**b**) threefold, or (**c**) twofold axes [183].

**Figure 13:**
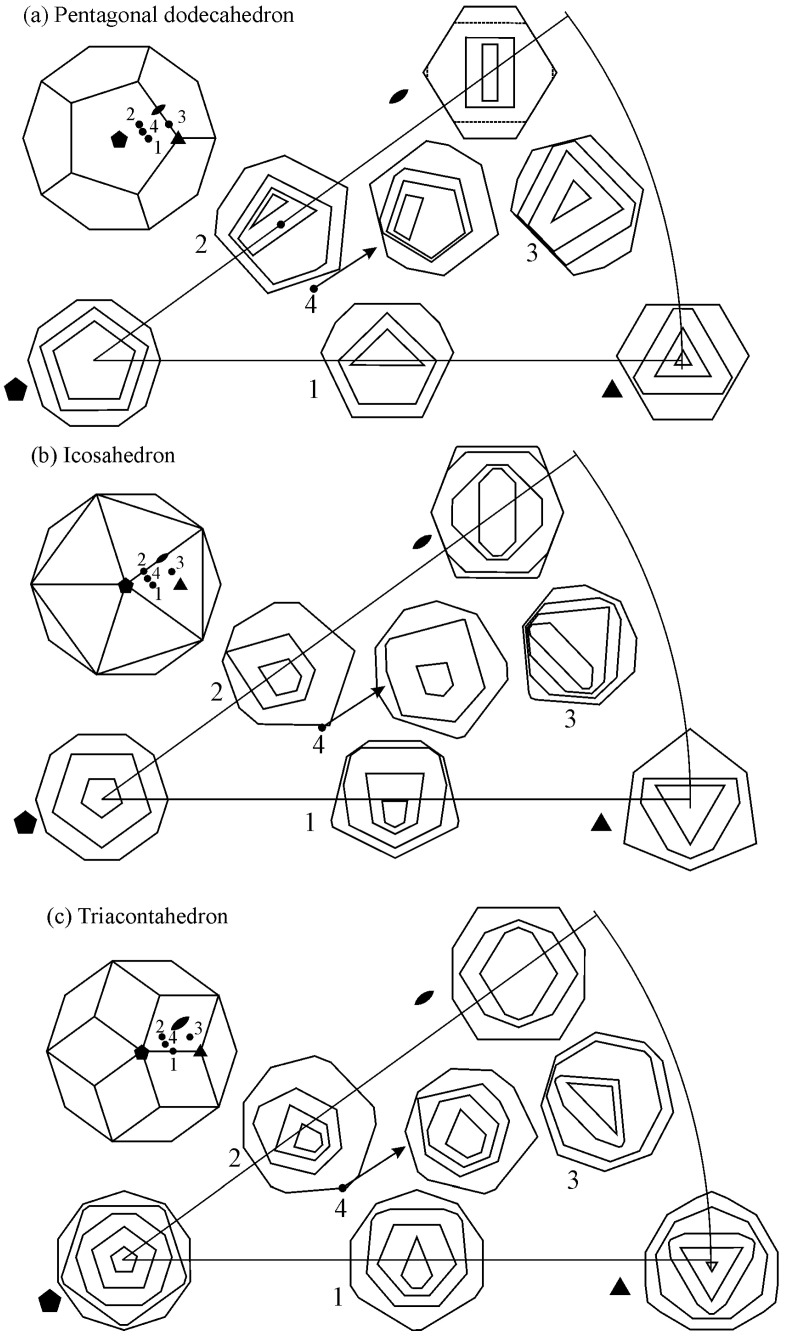
Presentation of possible 2D (**a**) pentagonal dodecahedron, (**b**) icosahedron, and (**c**) triacontahedron. Intersections at seven different quasicrystalline intersecting planes give many possible 2D shapes [183]. Numbers indicate the orientation of a quasicrystal; orientation 4 is indicated by the arrow.

To understand the shapes of particles in 2D, sections should be made through a pentagonal dodecahedron (Figure 12) and other possible shapes of primary icosahedral quasicrystals (Figure 13). In Figure 12, the intersecting planes are perpendicular to a pentagonal dodecahedron’s two-, three-, and fivefold axes. Intersections have different shapes, triangles, rectangles, pentagons, hexagons, octagons, and decagons, depending on where the metallographic plane intersects a polyhedron.

A quasicrystalline particle can have any orientation relative to the intersecting (metallographic) plane in a real material. It is impossible to investigate any orientation. We have selected seven different orientations and illustrated them within a stereographic triangle (Figure 13). The triangle’s vertices represent a fivefold, threefold, and twofold axis, and the possible intersections are the same as in Figure 12. In addition, the shapes along the four other axes were examined, which are indicated by numbers 1–4:

1. Between the fivefold and threefold axes;
2. Between the fivefold and twofold axes;
3. Between the twofold and threefold axes;
4. Within the stereographic triangle.

The analysis of all shapes showed that intersecting all polyhedra with a plane produces triangles, rectangles, pentagons, hexagons, octagons, and decagons, and their distorted and truncated variants. When a pentagon appears on a metallographic cross-section, it is impossible to state that a quasicrystal’s basic shape is the pentagonal dodecahedron. Several other characteristics should be considered when discriminating between different shapes.

At first sight, it is hard to determine the 3D shape of the particles in Figure 11 because they can be distorted due to the growth in the preferred directions, or their growth can be distorted by the presence of other particles. However, inspecting selected particles can eliminate some shapes, as shown in Figure 13. The trapezoidal shape, indicated by the arrow in (Figure 11b), seems possible only if the quasicrystal has the shape of a pentagonal dodecahedron. The pentagonal dodecahedron was observed in the same alloy [188,189]. This conclusion can be drawn when the specimen contains the IQC phase. Otherwise, faceted particles of crystalline phases can, in cross-section, have very different shapes, some closely similar to the shapes of quasicrystals [52].

**Figure 14:**
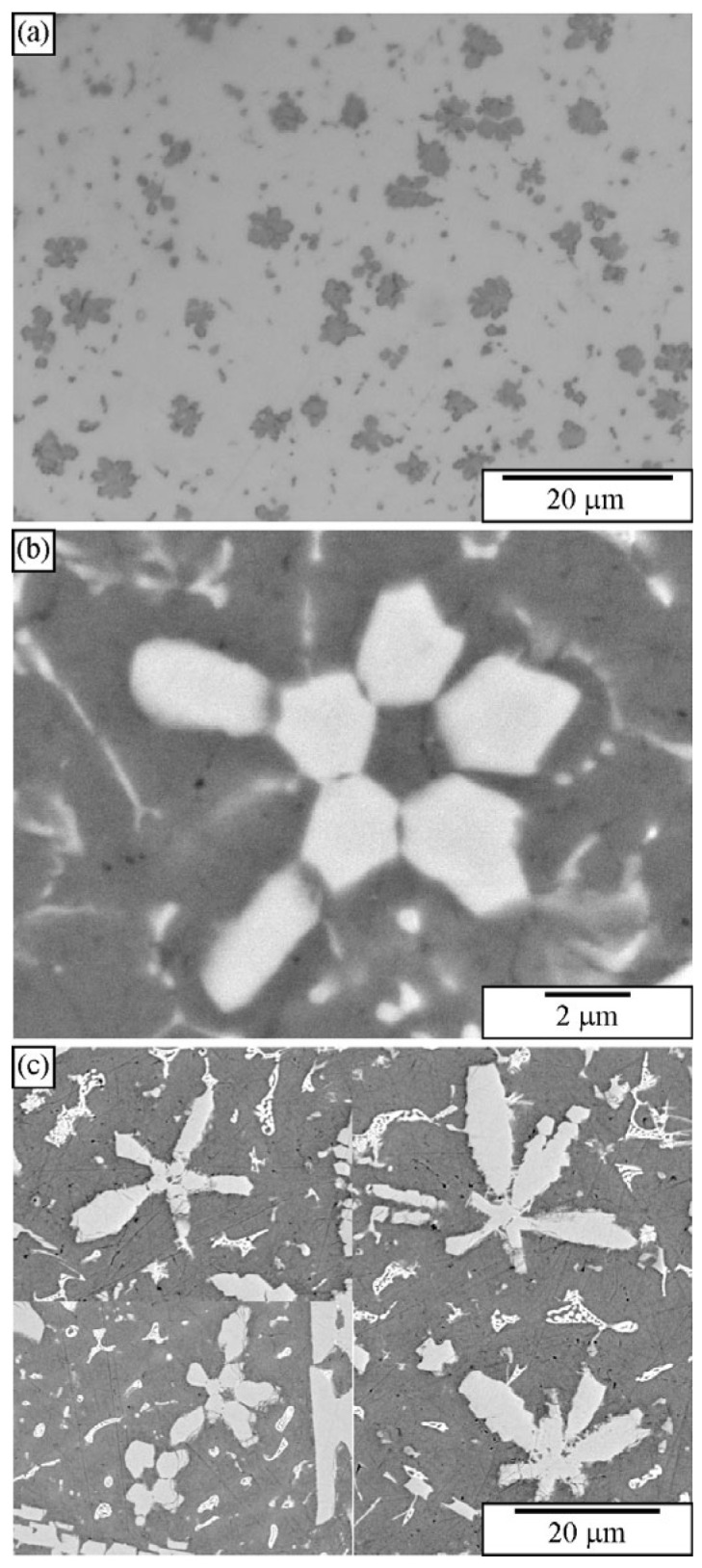
Quasicrystalline dendrites: (**a**) only small arms appear, (**b**) faceted dendrite arms, and (**c**) well-developed dendrite arms [186].

The growth of quasicrystals often takes place in preferred directions. As a result, dendritic arms can appear (Figure 14). Their maximum number is equal to the number of equivalent directions in the crystallographic system. The icosahedral system could have up to 12 fivefold, 20 threefold, and 30 twofold arms. The number of arms does not depend on the equilibrium shape of the icosahedral quasicrystal. The intersecting plane can cut one or more arms. The positions and dimensions of intersections depend on the orientation of the intersection plane, its distance from the centre of a dendrite, the shape of the dendrite arms, and the preferred growth directions. In the microstructure, differently developed dendrite arms can appear. In Figure 14a, short arms are shown. Nissen et al. [185] found faceted dendrites in the Al-Mn alloy; each dendrite arm was faceted and shaped like a pentagonal dodecahedron (Figure 14b). Otherwise, dendrite arms can show a slight tendency to faceting [148,157,158], with very long and well-developed dendrite arms (Figure 14c). A procedure for determining the preferred growth direction from a 2D cross-section is given in Ref. [183].

### 2.4. Identification of Quasicrystalline Precipitates in Al-Alloys

IQC precipitates can form in many Al-alloys. They precipitate from the supersaturated solid solution. Hansen and Gjonnes [190] found a metastable IQC phase in a heat-treated Al-Mn alloy, which subsequently transformed to the cubic α-AlMnSi phase. Li and Arnberg [191] discovered IQC dispersoids in a commercial 3003 alloy, which precipitated at the early precipitation stage. Quasicrystalline precipitates also formed during ageing in Al-Mn-based alloys fabricated by SLM [150]. Mochugovskiy et al. [192,193,194] discovered IQC precipitates in some Al-Mg-Mn alloys during annealing. Zhan et al. [195] studied nucleation and growth of quasicrystal-related precipitates in Al-Er-Fe alloys. Yang et al. [5] found quasicrystalline clusters in Al-Mg-Zn alloys. Quasicrystalline precipitates also occur in commercial Al-Cu-Li alloys [196,197]. Quasicrystalline precipitates also formed as one of the three strengthening precipitates in an Al-Zr-Gd(Yb) alloy [198]. Zr, Gd, and Yb formed L1_2_ precipitates, but the Fe and Si impurities formed IQC precipitates.

Partly coherent IQC precipitates predominantly formed on the dislocations and dislocation walls. IQC precipitates were also discovered in an Al_94_Mn_2_Be_2_Cu_2_ alloy during an in situ study of the temperature stability of the IQC phase formed during solidification [199]. They were found in samples heat-treated at 300 and 400 °C [200]. IQC precipitates in this alloy have a primitive icosahedral structure (space group $Pm\bar{35}$). They form at least a semi-coherent interface with an aluminium matrix, and both phases have a specific mutual orientation relationship. All aluminium fourfold axes are parallel to the three twofold axes of IQC, and at the same time, threefold aluminium axes are parallel to the threefold axes of IQC. Stan-Głowińska et al. [97,201] discovered nanoscale IQC precipitates in Al-Mg-Zn alloys containing Ga. Zhang et al. found that small amounts of iron in Al-V, Al-Mo, and Al-Cr alloys can give rise to a fine dispersion of P-phase precipitate at the grain centres. The P-phase is a quasicrystalline phase and has an orientation relationship with the Al matrix as i2\\[001]Al, i3\\[111]Al, and i5\\[t10]Al, t = (1 + square-root 5)/2 [202].

Icosahedral quasicrystalline precipitates can also form in Mg alloys [203,204], stainless steel [205], and Zr-amorphous alloy [206,207].

**Figure 15:**
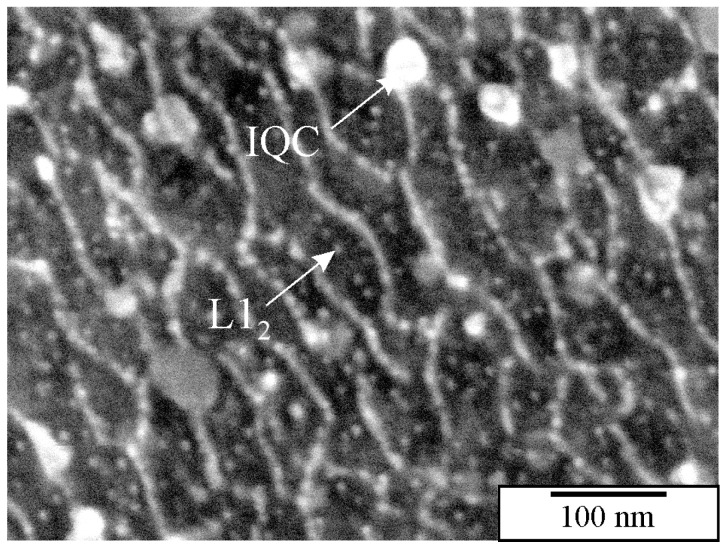
Quasicrystalline particles and L1_2_ precipitates in the α-Al matrix. Bright ridges are preparation artefacts that were not confirmed by TEM investigations. Mechanical polishing and etching using SiO_2_ and H_2_O_2_ were applied [208].

IQC and other quasicrystalline precipitates are relatively small, ranging from 10 to 100 nm. They can be thoroughly investigated in TEM. However, TEM samples are small, and the distribution of precipitates can be determined in a relatively small area. Thus, developing a metallographic procedure that enables observing as many tiny particles as possible in HR SEM is imperative. Österreicher et al. [162] developed a procedure for revealing the Mg_2_Si precipitates in Al-Mn-Si alloys using 30% nitric acid in methanol. The same method dissolved quasicrystalline precipitates in the authors’ laboratory. However, there is an ongoing investigation regarding the development of metallographic methods to identify small nanosized quasicrystalline particles. The preliminary findings using mechanical polishing with colloidal SiO_2_ and etching using diluted hydrogen peroxide, H_2_O_2_ show promising results (Figure 15). Colloidal silica with a granulation of 0.06 µm and 30% hydrogen peroxide in water with a ratio of 5:1 was used. Polishing with small abrasive grains and low surface tension removes the oxide layer and slightly embosses the surface. The procedure takes 1 to 2 min, with mandatory ultrasonic cleaning in distilled water without using cleaning alcohol. Quasicrystalline particles with a size of 30–50 nm and L1_2_ precipitates with a size of 10–20 nm can be seen. The method needs considerable improvement since several artefacts appear at the nanoscale (bright ridges in Figure 15) [208].

The second approach uses physical polishing and etching with Ar ions. The samples can be prepared by conventional mechanical polishing and finally exposed to Ar ions. Ion polishing improves the surface quality of metallographic samples by using broad-beam argon ion milling, which removes material at the atomic level without causing deformation or disturbance to the microstructure. This method is particularly effective for high-technology materials and complex assemblies, producing flat surfaces convenient for light microscopy, SEM, or EBSD [209,210,211].

**Figure 16:**
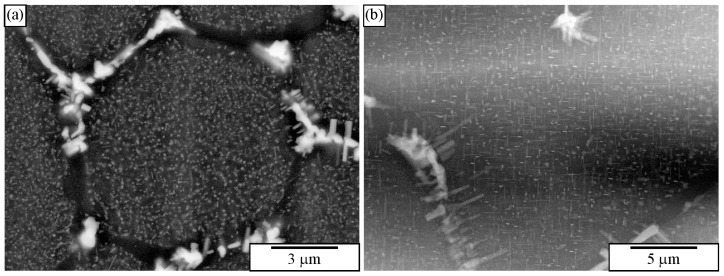
Comparison of HRSEM and TEM micrographs in an Al-Mn-Cu-Be-Sc-Zr alloy prepared by physical polishing and etching with Ar ions. (**a**) HRSEM and (**b**) TEM [208].

By changing operation parameters, such as accelerating voltage, sputtering angle, and duration, it was possible to prepare the surface with visible microstructural constituents without a deformation layer. Figure 16 compares the same alloy’s HR SEM and TEM micrographs, showing the extraordinary degree of resemblance. It is possible to recognise particles as small as 10 nm. In the actual sample, L1_2_ precipitates were present, with sizes around 5 nm, which could not be recognised despite some brighter areas in the α-Al matrix.

### 2.5. Alternative Methods for the Identification of Quasicrystalline Phases Using Metallography

Previous sections dealt with identifying quasicrystalline particles based on their morphology using light and scanning electron microscopy. SEM still needs an adequately metallographically prepared sample; however, the nature of interactions of electrons with the sample offers several ways to identify crystals.

The electron channelling effect in SEM is a phenomenon where the orientation of the crystal lattice influences the interaction of an electron beam with a crystalline material. When the electron beam is aligned with specific crystallographic planes (usually low index), the penetration depth can be significantly larger; thus, fewer electrons are backscattered. It increases the contrast in SEM images. The contrast level is weak, necessitating a minimum beam current of 5 nA for visibility [212]. It can enable the visualisation of crystallographic defects and crystal orientations.

Shindo et al. [213] found a strong electron channelling effect in a quasicrystal of an Al-Cu-Fe alloy, which has a near-perfect icosahedral symmetry and low phason density. When the electron beam was parallel with one of the high symmetry zone axes, such as the fivefold and twofold, the intensities of the characteristic X-ray of Fe and Cu relative to that of Al were much stronger than under non-channelling conditions. Thus, this method could identify quasicrystals in SEM’s without EBSD.

**Figure 17:**
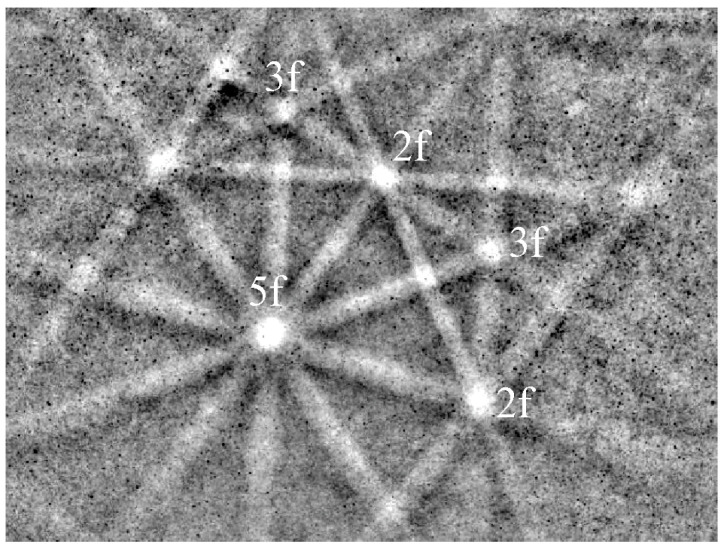
EBSD Kikuchi pattern of an icosahedral particle in an Al-Mn-Cu-Be alloy with indicated twofold (2f), threefold (3f), and fivefold (5f) poles [208].

EBSD (Electron Backscatter Diffraction) is an advanced microstructural characterisation technique used in SEM to determine the crystallographic orientation of materials. It is especially powerful for studying crystalline solids at the microscale. A polished crystalline sample is tilted at a high angle (~70°) inside an SEM chamber. The electron beam interacts with the tilted surface, causing backscattered electrons to diffract. These diffracted electrons form a pattern of lines called a Kikuchi pattern on a phosphor screen. The pattern is recorded and analysed to determine the crystal orientation at that specific point (Figure 17). By scanning across an area, EBSD can build up orientation maps that show the crystallographic structure of the sample at high spatial resolution, typically for bulk samples above 100 nm.

EBSD has often been used to characterise quasicrystals. A systematic orientation analysis for icosahedral and decagonal quasicrystals was provided by Winkelmann et al. [214]. Labib et al. [215] used EBSD to characterise IQC and quasicrystalline approximant in Cd-Mg-RE (RE = Y, Sm, Gd, Tb, Dy, Ho, Er, Tm) systems. Kikuchi patterns revealed a high resemblance between the two structures. Winkelmann et al. [214] mapped the locally varying orientations in samples of icosahedral quasicrystals observed in a Ti40Zr40Ni20 alloy and AlNiCo decagonal quasicrystals. Tanaka et al. [216] developed a new analysis system for identifying the orientation of an IQC in an SEM with an EBSD instrument. The system was successfully applied to determine the orientation of any given IQC grain by reading and assigning the Kikuchi bands obtained with the EBSD. Naglič et al. [217] identified IQC and DQC in Al-Mn-Ga alloys. The quasicrystalline phases larger than 100 nm could be identified using bulk specimens, not nanoscale precipitates.

Recently, a new approach to SEM-based diffraction has been developed [218,219]. It is called transmission EBSD (t-EBSD) or transmission Kikuchi diffraction (TKD). TKD improves spatial resolution, enabling effective characterisation of nanocrystalline materials because resolution could be below 10 nm [220,221]. The final spatial resolution depends on the sample atomic number, beam energy, sample thickness, and tilt angle. TKD samples can be prepared using the standard transmission electron microscopy preparation (TEM) methods. Electropolishing 3 mm diameter metallic discs is often the most effective because it can produce a large electron-transparent area. However, FIB-SEM can also be used. The optimum thickness depends on the material and lies between 50 and 100 nm [222].

Transmission scanning electron microscopy on the TKD samples allows microchemical analysis at a very high spatial resolution. In conventional energy-dispersive spectroscopy/scanning-electron microscopy (EDS/SEM) analysis, the pear-shaped interaction volume of incident electrons in the bulk sample determines spatial resolution; thus, the actual scope of EDS analysis is usually a few cubic microns. Lowering the accelerating voltage can effectively reduce the interaction volume of the electron beam. Liu et al. [223] used this method to further simulate EDS mapping of Ni nanoparticles.

Atom probe tomography (APT) is an advanced microscopy technique that allows 3D reconstruction of a material at near-atomic resolution and elemental identification of each atom. Quasicrystalline precipitates were analysed with this technique by Farkoosh et al. [224] in an age-hardened and creep-resistant Al-0.5Mn-0.3Si (at.%) alloy by Sn inoculation. The nanoscale quasicrystalline precipitates were also revealed in a Mg–6Zn–4Al–1Sn–0.5Mn alloy [225].

## 3. Deep Etching and Particle Extraction

The icosahedral quasicrystalline particles rarely exhibited a pentagonal symmetry on the polished surface [183]. It is even harder for the decagonal quasicrystals that mainly appear as platelike particles [70], which can be found in many crystal systems. Consequently, it is challenging to identify quasicrystalline particles only from 2D sections prepared by traditional metallographic techniques. Therefore, there is a great interest in developing methods for obtaining 3D microstructures, especially when explaining the morphology evolution at different processing techniques [226,227]. The commonly used methods are deep etching and particle extraction from the aluminium matrix [228]. Many researchers have dealt with the methods of deep aluminium etching [143,228,229,230]. A review article on this topic was published by Gupta et al. in 1996 [228]. In the article, they collected the methods and etchants known at that time. It is frequently desirable that the Al matrix be removed in a controlled way [231]. In the first stage, the surface oxide layer should be removed or at least considerably thinned by mechanical and electrochemical polishing or electrolysis. The Pourbaix diagram for the system Al-H_2_O at 25 °C shows that the oxide layer is stable for a pH between 5 and 8.5 [232], and this range is to be considered by the selection of the solution for chemical removal of the oxide layer.

During deep etching, the matrix should be removed around the particles, while the particles should be retained unchanged. In addition, they remain at their original position. Less material must be removed by deep etching than by particle extraction. When the etching depth approaches the typical particle sizes, deep etching can be stopped because the morphology of particles becomes visible [143]. Longer etching times are required when revealing larger particles in the microstructure. It is thus extremely important that a reagent does not dissolve quasicrystalline and other phases during particle extraction so that they still exhibit their original shape after removing the matrix [233]. The particles should be cathodic relative to the matrix. In the opposite case, if the particles are anodic, they will dissolve, leaving a cavity at the site of the particle [185]. Table 4 gives characteristics of chemical extraction methods for deep etching and extraction of particles, mainly in the aluminium alloys.

The first attempt to reveal the quasicrystalline phase using deep etching in rapidly quenched Al-5.3 at.% Mn alloy was performed by Csanady et al. [170]. They used a slightly acidic medium (the composition is not given in the article), which nicely preserved the crystalline Al_6_Mn and Al_4_Mn particles; however, the icosahedral quasicrystalline phase completely dissolved. Nissen et al. [185] studied two rapidly solidified alloys with the nominal compositions Al_6_Mn (14.3 at.% Mn) and Al_17_Mn (5.6 at.% Mn). The melt-spun ribbons were etched in a 250 mL methyl alcohol solution with 2 wt.% KI. The alloy sample was suspended in the centre of a 400 mL beaker containing the etchant and surrounded by a high-purity Al or Pt cathode. The etching lasted one hour, during which the voltage applied was 2–5 V and the current was 0.1–0.3 A. This etching process resulted in an almost complete isolation of all quasicrystalline particles from the surrounding crystalline Al solid solution matrix of Al. They also electropolished melt-spun ribbons at −20 °C in a solution consisting of a mixture of perchloric acid (HClO_4_) and 20% methanol. After this treatment, the quasicrystal particles dissolved and appeared as cavities in the Al-rich matrix.

Kang et al. [226] determined the icosahedral quasicrystalline phase’s 3D morphology and formation process in rapidly solidified Al–Mn alloy. The ribbons were electro-etched in a 250 mL methyl alcohol solution with 2 wt.% KI. The voltage, temperature, and current density for electrolytic etching were 10 V, 25 °C, and 0.1 A/cm^2^, respectively. They revealed the morphology of the quasicrystalline phase in melt-spun ribbons.

Kang et al. [234] used a NaOH aqueous solution to dissolve the eutectic matrix and preserve the primary IQC particles in the Al–6Mn–2.5Be alloy. They did not give the exact composition of the etchant. However, they revealed the three-dimensional morphological evolution and growth mechanisms of primary IQC particles during directional solidification. On the contrary, Bončina et al. [201] did not reveal the quasicrystalline phase in an Al-Mn-Be-Cu alloy using 5% aqueous solution of NaOH for 30 s. The presence of Cu in the IQC phase may change the potential of the IQC phase relative to the matrix.

Wang et al. [235] deep etched AZ magnesium alloy using 10% nitric acid in ethanol for 5–10 min. They could dissolve the magnesium-rich matrix to reveal an Al-Mn-based decagonal quasicrystal and some other crystalline phase.

Zhu et al. [167] found that a single icosahedral quasicrystal can be obtained in the Mg-Al-Zn alloys. The icosahedral quasicrystals possessed three morphologies: well-developed dendrites and coral-like and petal-like quasicrystals. Samples were mechanically polished and etched in a 5% nitric acid solution and 95% ethanol.

**Figure 18:**
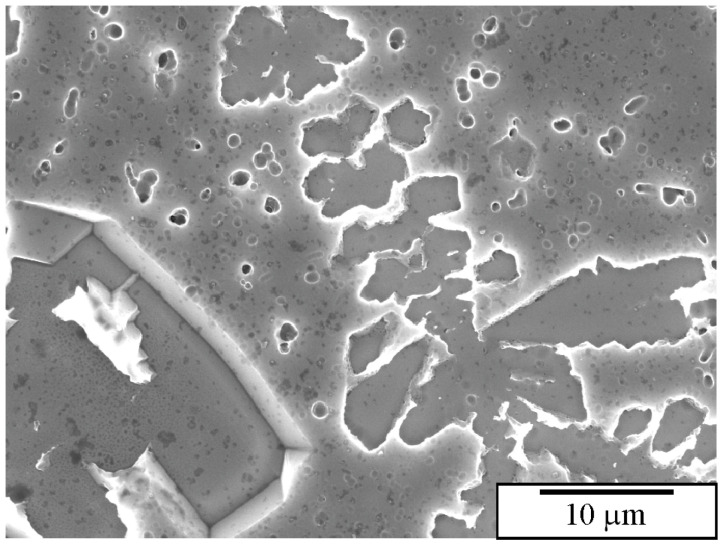
The scanning electron micrographs of microstructures after 30 s etching with the etchant consisting of 2.5 mL of HCl, 5 mL of HNO_3,_ and 1 mL of HF in the as-cast alloy Al_86_Mn_3_Be_11_ after vacuum melting and slow cooling during solidification [236].

Bončina et al. [236] tested several etchants to reveal the morphology of quasicrystals and other phases in Al-Mn-Be and Al-Mn-Be-Cu alloys. They found that a solution of strong acids (2.5 mL HCl, 5 mL HNO_3,_ and 1 mL HF) slightly attacks the Al matrix, while intermetallic phases were attacked much more strongly (Figure 18). The IQC phase was dissolved entirely, and only its silhouette remained (Figure 18). This indicates that the IQC and other intermetallic phases acted as anodes and the Al matrix as a cathode.

**Figure 19:**
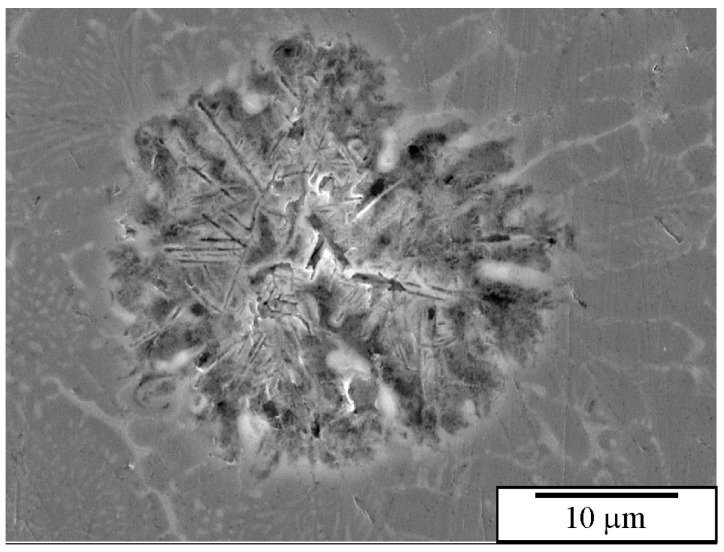
The scanning electron micrographs of the microstructure of alloy Al_94_Mn_2_Be_2_Cu_2_ with a footprint of the primary quasicrystalline particles after etching for 30 s in a solution of 5 mL of HNO_3_, 2.5 mL of HCl, and 70 mL of alcohol [236].

Dilute solutions made of HCl and HNO_3_ have been extensively used to etch aluminium alloys deeply [228,231]. When utilised to etch the aluminium quasicrystalline alloys containing higher volume fractions of intermetallic phases, etching with a solution of 5 mL of HNO_3_, 2.5 mL of HCl, and 70 mL of alcohol for 30 s resulted in almost complete dissolution of quasicrystalline particles, and the aluminium matrix remained intact (Figure 19) [236].

Bončina et al. [237] give special attention to improving the procedure of dynamic deep etching and particle extraction from aluminium alloys, especially dedicated to quasicrystalline particles. The basis represents the iodine methanol solution (10 g of iodine and 25 g of tartaric acid in 250 mL of methanol; the ratio of iodine to metal should be greater than 4.7), which was developed by Guzowski et al. [230] to determine the morphology of grain-refining particles in Al-Ti-alloys. The process is not straightforward for aluminium alloys containing quasicrystals; thus, it was modified [237]. The main characteristics of the modified process are presented in more detail below.

There are two main problems. Firstly, the process does not start just by immersing samples into a solution; secondly, iodine or CuI remains in the sample after the process. Several steps should be taken to obtain excellent results. An aluminium sample is initially rinsed into a solution containing 3 to 10 g of iodine and 10 to 17 g of tartaric acid in 100 mL of methanol and put into an ultrasonic cleaner. The sample is sequentially exposed to ultrasonic vibrations and resting, lasting from 10 min up to 24 h. The sequence of rinsing and dissolving, followed by exposure to ultrasonic action, should be alternately repeated 10 to 50 times. After deep etching, the sample is put into the ultrasonic bath to remove the reaction products, washed with alcohol, and dried. The sample is then broken to expose a clean fracture surface with a partly removed aluminium matrix and undissolved particles.

The solution was filtered using a glass filter to obtain extracted particles. The filter retained particles larger than 300 nm. The water-soluble iodides are dissolved by washing the sediment in alcohol and water. The smaller nanoparticles can be collected from the clear alcohol–water solution by the grid and air drying.

**Figure 20:**
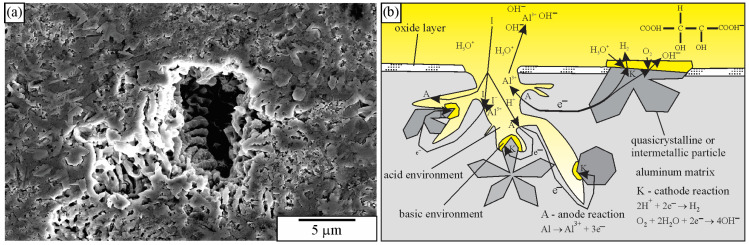
Characteristic of deep etching in iodine-methanol solution. (**a**) The scanning electron micrographs of the hole on the surface of the sample. (**b**) A schematic presentation of the deep etching of an aluminium alloy, which contains an intermetallic phase [236]. Arrows indicate the movements of the ions.

The etchant composition is influential, but implementing all etching stages is more critical. The oxide surface layer is not removed, but the ultrasonic vibrations cause a local disruption of the oxide layer (Figure 20a), enabling the penetration of the etchant into the sample (Figure 20b).

**Figure 21:**
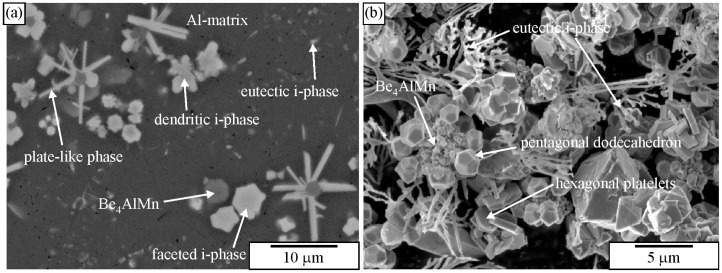
Microstructure of an Al-Mn-Be alloy in the as-cast condition. (**a**) Backscattered electron micrograph of the polished sample before etching and (**b**) secondary electron micrograph after etching with an iodine-methanol solution [236].

Figure 21 shows images of an as-cast Al-Mn-Be alloy before and after etching with an iodine-methanol solution. The alloy was exposed to the solution for several days. It is evident that with this solution, all phases present in the microstructure were observed. It seems that only Be_4_AlMn was partly dissolved. A fine, branched structure of the quasicrystalline eutectic phase also remained unchanged.

**Figure 22:**
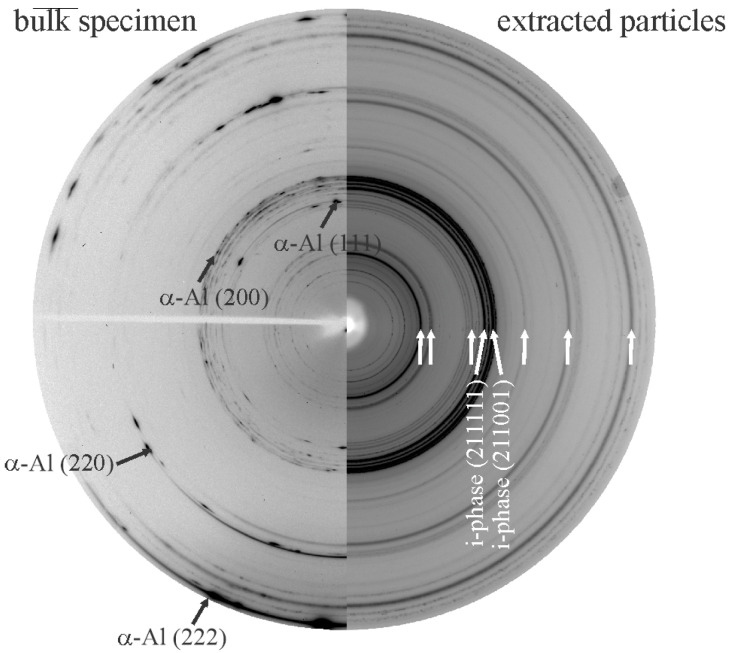
Two-dimensional diffraction patterns of a multiphase Al-Mn-Be alloy in the as-cast condition after using synchrotron radiation. (Left) In the bulk specimen, the powder pattern shows incomplete Al rings, indicating a small number of Al grains, and the rings of other phases are relatively weak. (Right) Extracted particles. The Al-rich matrix is dissolved, and lines of minor phases, indicated by the arrows, become more pronounced. Only the most significant rings of the IQC phase are indicated [233].

Figure 22 shows 2D diffraction patterns of an Al-Mn-Be alloy in the as-cast condition. It was also confirmed that all reaction products formed during dissolution were removed after applying the iodine-methanol solution. There were no rings corresponding to iodine or other iodides. Removing the Al matrix results in much better visibility of rings of minor phases. In this case, rings belonging to IQC become visible and allow unambiguous identification.

### 3.1. Deep Etching of Melt-Spun Ribbons

Melt spinning enables the study of equilibrium shape and the roughening behaviour of the quasicrystals. The rapid solidification of dilute Al-Mn-Be and Al-Mn-Be-B alloys makes possible substantial undercooling, thus promoting nucleation of the quasicrystal in the melt [11]. Later, the Al grains nucleate, and their very fast growth enables the trapping of small quasicrystals.

**Figure 23:**
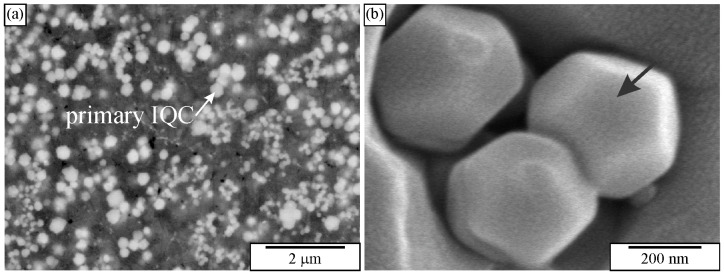
Quasicrystals in melt-spun ribbons of an Al-Mn-Be-B alloy. (**a**) Backscattered electron image before deep etching. The micrograph is similar to Figure 9. (**b**) Secondary electron image of deep-etched quasicrystalline particles using an iodine-methanol solution. Quasicrystalline particles with the shape of the pentagonal dodecahedron, the arrow indicates the pentagonal facet [227].

Figure 23a shows a microstructure typical of 30–90 μm thick ribbons. This is an enlarged image of Figure 9. These ribbons possessed a relatively uniform distribution of tiny spherical quasicrystalline particles with sizes between 50 nm and 500 nm in diameter in an α–Al matrix. Backscattered electron images indicated that some particles had spherical shapes, while some showed a tendency for faceting. However, the resolution was usually too low to determine their shape precisely.

During deep etching with the iodine-methanol solution, the matrix was removed around particles, revealing their pentagonal dodecahedron shape. On the particle indicated by the arrow, pentagon facets can be easily recognised. Thus, SEM of deep-etched melt-spun ribbons unambiguously revealed the shape of faceted IQC particles [227].

**Figure 24:**
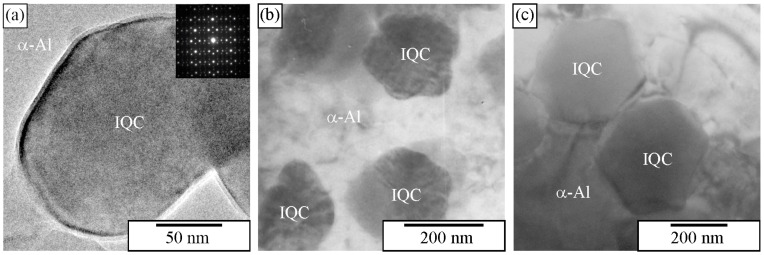
IQC particles in melt-spun ribbons (bright-field TEM micrographs). (**a**) Extracted particles of the IQC phase in the alloy Al-Mn; the image was taken along a twofold axis; (**b**) bright-field micrograph of the alloy Al-Mn-Be; and (**c**) bright-field electron micrograph of the alloy Al-Mn-Be-B; the image was taken along a twofold axis [227].

**Figure 25:**
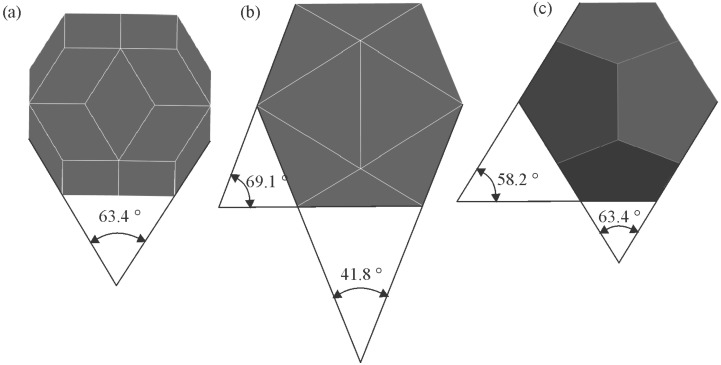
Projection of the (**a**) triacontahedron, (**b**) icosahedron, and (**c**) pentagonal dodecahedron along their twofold axis in TEM. The projections are considerably different; thus, it is possible to determine the shapes of icosahedral quasicrystalline particles in TEM [227].

Transmission electron microscopy allows the determination of quasicrystalline shapes. We can use foils or extracted particles. Figure 24a shows that IQC particles with a size of less than 100 nm ribbons had rounded edges. Particles with sizes up to 200 nm can be almost ideal spheres, while some have bumps (Figure 24b). A particle in Figure 24c is faceted. By comparison of the projection with Figure 25, it is clear that the particle has the shape of a pentagonal dodecahedron, which was observed in SEM after deep etching.

**Figure 26:**
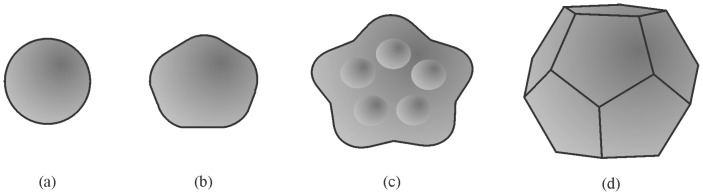
The effect of undercooling on shapes of IQC particles in melt-spun Al-Mn-Be-B ribbons: (**a**) sphere, (**b**) particle with rounded edges, (**c**) particle with bumps (initiation of dendritic growth), and (**d**) pentagonal dodecahedron. Shape (**a**) is mainly present at the wheel side, while shape (**d**) can be observed at the air side, where the cooling rates are lower [227].

The possible shapes of IQC particles formed upon the primary crystallisation of melt-spun ribbons are shown in Figure 26. The evolution of shapes depends on the undercooling. A larger undercooling can be obtained at the wheel side, resulting in spherical particles because of dynamic roughening [164]. With the decreasing cooling rate, the particles possess some flat faces with rounded corners. Constitutional undercooling can lead to morphological instability of the solid–liquid interface, resulting in bump formation. Such development can lead to the formation of large dendrites with rounded arms. At a smaller undercooling, the effect of dynamic roughening diminishes, resulting in the formation of almost ideal pentagonal dodecahedra.

### 3.2. Deep Etching of Alloys with Polygonal and Dendritic Icosahedral Quasicrystals

**Figure 27:**
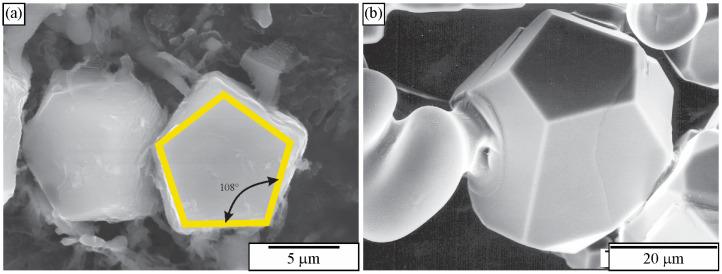
Secondary electron micrographs of IQC particles with pentagonal dodecahedral morphology are presented in Figure 10b. (**a**) Alloy Al-Mn-Be, the yellow regular pentagon fits on the particle facet, and the ideal angle between two pentagon edges, and (**b**) alloy Al-Cu-Fe [188].

**Figure 28:**
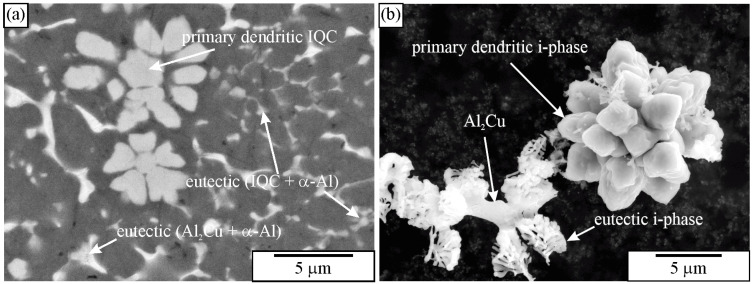
Microstructure of the alloy Al_94_Mn_2_Be_2_Cu_2_ in the as-cast condition. (**a**) A backscattered electron micrograph shows IQC dendrites and two-phase (IQC + α-Al) and (α-Al + Al_2_Cu) eutectics. (**b**) Secondary electron micrograph of extracted primary IQC, rod-like eutectic IQC, and eutectic Al_2_Cu phase [186].

Figure 27a shows an IQC quasicrystal after deep etching in an as-cast Al-Mn-Be-Cu alloy. The backscattered electron image of the same sample is given in Figure 10b. Figure 28b shows an IQC quasicrystal after deep etching in an as-cast Al-Cu-Fe alloy. In both cases, the shape of the IQC was a pentagonal dodecahedron.

Figure 28 shows IQC dendrites. Recognition of the quasicrystalline nature is apparent in the extracted sample.

**Figure 29:**
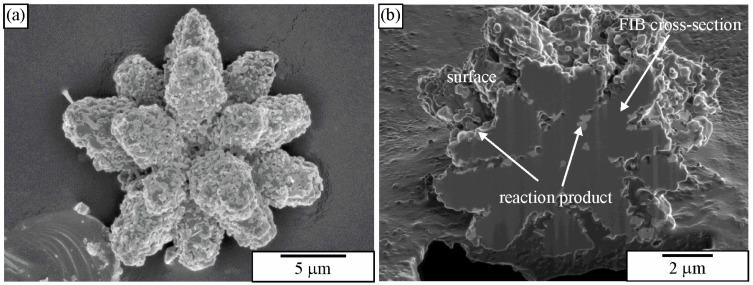
Extracted icosahedral dendrite with the arms in threefold directions. (**a**) A secondary electron SEM micrograph of an extracted quasicrystalline particle (alloy Al-Mn-Be-Cu after annealing at 500 °C for 5 h) is covered by reaction products. (**b**) Extracted quasicrystalline particle after FIB cross-sectioning (ion-induced secondary electrons). Internal structure is revealed, with the particles of a new phase appearing on the surface of the quasicrystalline particle and inside it [233].

Figure 29a shows an extracted icosahedral quasicrystalline dendrite covered with small particles. Its shape can be recognised immediately, indicating the strength of the particle extraction technique. On the other side, there is no indication of how deep the particles penetrate the quasicrystal. This information can be gathered from 2D metallographic sections. In our time, the entire quasicrystal can be sectioned with FIB (Figure 29b). Thus, one can simultaneously see the phases’ morphology and internal structure. Making FIB cross-sections of the extracted particles can be much faster than making cross-sections in bulk material since much less material must be removed.

**Figure 30:**
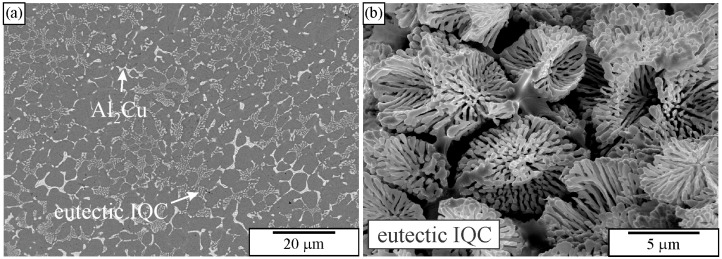
Alloy Al_94_Mn_2_Cu_2_Be_2_ phase in the as-cast condition. (**a**) Backscattered electron micrograph of the polished specimen. (**b**) Secondary electron micrographs of the colonies of the eutectic icosahedral quasicrystalline phase [233].

The IQC was also a part of two-phase eutectic cells (α-Al + IQC). Extraction of particles revealed the rodlike structure of the eutectic IQC phase (Figure 30). The rods evolved from a common centre. As the distance from the centre increased, frequent branching occurred. Branching keeps the distance between branches as constant as possible. Detailed TEM analysis of the eutectic IQC showed that the whole rodlike structure is a single quasicrystal. No sharp orientation changes were observed, not even at branching positions. This indicated that branching did not occur by, e.g., twinning, which is typical for the Si phase in the Al-Si eutectic. Still, growth direction can be quickly changed to one of the other preferred directions due to the very high symmetry of the icosahedral phase. Furthermore, it was observed that orientation was changing uniformly along each rod. A rotation of 2.7° around a fivefold zone axis occurred when moving from position 1 to position 2. This kind of lattice rotation might indicate the presence of quasilattice defects, such as phason strains [238]. This was confirmed by peer examination of the diffraction patterns since many diffraction spots were deflected from their ideal positions (significantly weaker ones), and the shapes of some spots showed strong anisotropy.

**Figure 31:**
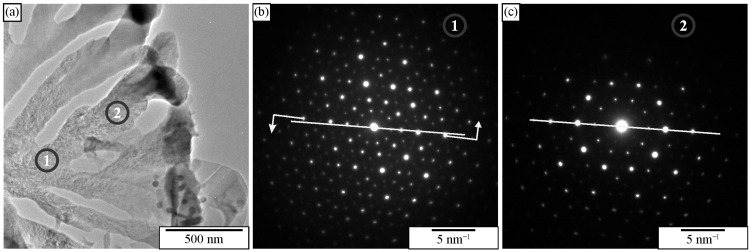
Eutectic IQC extracted by iodine-methanol solution. (**a**) The bright-field micrograph in TEM. (**b**) Selected area diffraction patterns taken along a fivefold axis at position one are indicated in (**a**). Arrows indicate the rotation direction of the diffraction patterns when moving from point 1 to 2. (**c**) Selected area diffraction patterns taken along a fivefold axis at position 2 indicated in (**a**) [186].

Particle extraction can also provide advantages by applying characterisation techniques other than SEM-related ones. For instance, the extracted particles can be reliably examined with TEM. Thin particles are placed on a holey carbon grid, allowing TEM examination without other preparation steps (Figure 31). Also, HRTEM and analytical examinations, such as EDS and EELS, can be performed on the extracted particles [186].

### 3.3. Alternative Methods for Determining 3D Morphologies of Quasicrystals

The first large stable quasicrystals were obtained by slow growth of the quasicrystals from the melt. During solidification, the melt disappeared, leaving holes in which the grown quasicrystals were present. The morphology of the quasicrystal was revealed without any need for metallographic preparation. This way, Beeli and Nissen [184] determined the growth morphology of icosahedral Al-Mn-Pd single quasicrystals. Al-Fe-Cu single crystals can also obtain their equilibrium shape in the cavities during heat treatment [188]. These are special cases that can be applied to stable quasicrystals.

Kral et al. [239] obtained 3D shapes of phases in steels using computer-aided visualisation of 3D reconstructions from serial section images. They gradually removed approximately 0.2 μm of material step-by-step metallographically and took photos of the section after each step. This is rather a slow procedure, which is used rarely. No such attempt was reported in connection with quasicrystals.

Nowadays, sequential cross-sectioning is performed using FIB (usually dual-beam SEM/FIB systems are used). FIB-SEM tomography has proven to be a mature and reliable technique, which can be applied to different materials and research fields [240]. A FIB/SEM system combines focused ion beam milling with high-resolution electron imaging to prepare, image, and analyse cross-sections of materials. It is possible to obtain detailed morphological, compositional, and crystallographic information with spatial resolution in the nanometre range (1–10 nm for imaging; ~10–20 nm for chemical mapping). A cross-section is produced by FIB milling. After FIB removes a slice of material, the electron beam scans the surface. Secondary electrons (SEs) and backscattered electrons (BSEs) provide high-resolution topographic and compositional contrast. The electron and ion beams are arranged at a fixed angle, enabling simultaneous imaging and milling of cross-sections. FIB can reveal a subsurface microstructure, which is imaged by SEM at the same time. By sequentially milling thin slices with FIB and imaging each slice with SEM, a 3D reconstruction of the internal microstructure can be created. Lasagni et al. [241] used FIB-SEM tomography to characterise as-cast and solution-treated AlSi12(Sr). Singh et al. [242] determined nanoscale precipitates in an AA7075-T651 alloy. However, no reports have been found regarding the shape of quasicrystalline phases.

It would be possible to obtain the morphology of large quasicrystalline particles by 3D reconstruction with sub-micro or micro-CT imaging or 3D X-ray microscopes. Both methods require expensive equipment, which is becoming increasingly more available but is often time-consuming. In most cases, these methods do not reach the necessary resolution for metallography [243]. X-ray microscopy (XRM) is very promising in materials science. X-ray microscopy is a technique with the resolution between optical and electron microscopy, offering substantial penetration depths that enable non-destructive imaging of thick and complex specimens. Spatial resolution spans from sub-10-nanometre scales achievable through advanced synchrotron-based methods to sub-micrometre levels in full-field and structured illumination approaches [244]. The characteristics depend highly on the X-ray energy range used; soft X-rays facilitate higher spatial resolution but with limited penetration [245], whereas hard X-rays allow imaging of thicker samples at modestly coarser resolutions. Depth of field is a significant strength of X-ray microscopy. X-ray microscopy enables several contrast mechanisms (absorption contrast for elemental and chemical sensitivity, phase contrast for visualisation of weakly absorbing specimens) while fluorescence and diffraction contrasts give chemical or magnetic information.

Atom probe tomography works by field-evaporating atoms one by one from a sharp needle-shaped specimen, identifying them using time-of-flight mass spectrometry, and reconstructing their original positions into a 3D atomic map. The specimen is prepared into a very sharp needle shape with a tip radius of ~50–100 nm using focused ion beam (FIB) or electrochemical polishing. Then a strong electric field (tens of V/nm) is applied to the tip, and short voltage pulses or laser pulses are used to trigger evaporation in a controlled way. The evaporated ions are accelerated toward a position-sensitive detector. Their time-of-flight (TOF), i.e., the time to reach the detector, depends on the ion’s mass-to-charge ratio. The original position of each atom on the tip is reconstructed using the detector impact position and the evaporation sequence. A 3D atom-by-atom map of the sample is created by combining millions of such evaporation events. APT has a spatial resolution of ~0.2–0.3 nm in depth and ~0.3–0.5 nm laterally. It can detect elements at a ppm-level concentration. It can analyse up to ~100 million atoms in a single dataset. However, conductive or laser-assisted evaporation is required for insulating materials, and the needle shape restricts sample geometry. APT can provide shapes of clusters and nanosized quasicrystalline precipitates [224].

## 4. Conclusions

This review considers different aspects of the metallography of quasicrystals, mainly focusing on the classical metallographic procedures and discussing alternative methods.

Much information about quasicrystalline phases can be obtained using light and scanning electron microscopy on metallographic samples (2D cross-sections of the microstructure). Adequately oriented quasicrystalline particles can show their characteristic shapes (pentagonal shape for icosahedral quasicrystals) or specific growth angle of eutectic quasicrystals. The possible shapes for faceted and dendritic icosahedral quasicrystals have already been presented in a systematic way. However, there is a research perspective to make similar systems for other types of quasicrystals.

For metallographically prepared samples, the application of EBSD provides information regarding the crystal structure and orientation of crystals. Although there are existing mathematical models that enable identification of quasicrystals and their orientation according to their Kikuchi lines, the main producers of EBSD equipment still do not have quasicrystals in their databases. Thus, the quasicrystalline phase is still not automatically identified, which makes its identification difficult for users who are not familiar with quasicrystals. However, for the skilled users, geometrical, chemical, and crystallographic information of particles larger than 100 nm can be obtained.

Hence, there is still an open question of whether the observation and reliable identification of quasicrystalline particles smaller than 100 nm on bulk samples is viable. The topic is not important just for quasicrystals but also for other phases. There is a research task of how to find an appropriate etchant that would uniformly remove a few nanometres of the matrix, exposing nanometre-sized particles.

Transmission scanning electron microscopy at low voltages (in typical SEM instruments) on thin lamellas (50–100 nm), prepared by FIB instruments, represents a possibility for geometrical, chemical, and crystallographic characterisation of nanoscale precipitates (10 nm or even less). Combined with transmission Kikuchi diffraction, it can also provide crystallographic information. However, it is not a standard method yet. Thus, advancing this method should be an important research direction.

Deep etching and particle extraction provide an excellent possibility of revealing quasicrystals’ morphology and their identification. In this area, the iodine-methanol solution was effective in some alloys. Here, there is still an open question of whether this method is applicable to other alloy systems. It is also rather slow; therefore, more systematic work in improving its behaviour is required. In the field of metallography, the selection of etchants is still mainly empirical. There is a lack of theory that can predict the phase dissolution in multi-phase alloys and especially forecast the retention of their 3D shapes.

Alternative methods that can reveal the 3D shapes of quasicrystals are rather rarely used (FIB/SEM 3D reconstruction, microCT, X-ray microscopy, and APT), so their more frequent application could reveal finer details in the shape and structure of quasicrystals that can disclose additional information regarding their formation and growth mechanisms.

Nevertheless, the most important method for analysing quasicrystalline structures, especially at the atomic resolution, will remain analytical TEM, providing reliable information on the detailed quasicrystalline structure, defects, composition, and geometrical properties.

## Figures and Tables

**Table 1:** Structure of selected quasicrystals. The dataset of more than 1400 entries can be downloaded from reference [55].

Alloy System	Quasicrystal Type
Al_86_Mn_14_	icosahedral
Al_4_Mn	decagonal
Al_70_Mn_10_Pd_20_	icosahedral
Al_70_Mn_15_Pd_15_	decagonal
Al_3_Mn_82_Si_15_	octagonal
Al_65_Cu_25_Fe_5_V_5_	dodecagonal
Al_55_Si_25_Mn_20_	icosahedral
Al_63_Cu_25_Fe_12_	icosahedral
Al_54_Pd_30_Yb_16_	icosahedral
Al_70_Ni_20_Rh_10_	icosahedral
Al_6_Li_3_Au	icosahedral
Al_70_Pd_20_Tc_10_	icosahedral
Al_73_Pd_18_Re_9_	icosahedral
Al_92_Fe_3_Cr_2_Ti_3_	icosahedral

**Table 2:** Some typical properties of quasicrystals. The dataset of more than 1400 entries can be downloaded from reference [55].

Property	Typical Characteristics	Reference
Electrical conductivity	Extremely low: ~10^−3^ to 10^−5^ Ω^−1^·cm^−1^ (semiconductor-like)	[136]
Thermal conductivity	1–2 W·m^−1^·K^−1^—low due to phonon scattering	[119]
Elastic moduli	Young’s modulus: 60–140 GPa	[137]
Hardness	Vickers hardness: 5–9 GPa	[138]
Friction coefficient	Extremely low: ~0.05	
Thermoelectric power (Seebeck coefficient)	Up to 200–400 µV/K	[119]
Internal friction	Low at T < 4 K; resembles amorphous solids	[137]
Corrosion and oxidation resistance	High, similar to stainless steel	[139]
Magnetic properties	Low concentrations of magnetic atoms (e.g., Mn, rare-earth elements) and spin-glass behaviour are due to the random distribution of localised moments Competing Kondo and RKKY interactions without long-range magnetic order. Unusual antiferromagnetic correlations influenced by aperiodicity	[140]
Fracture behaviour	brittle	[141,142]

**Table 3:** The list of most used etchants by characterisation of quasicrystals in Al-alloys.

Etchant	Etching Conditions	Reference
Keller’s reagent (1 mL of HF, 1.5 mL of HCl, 2.5 mL of HNO_3_, 95 mL of distilled water)	Room temperature, 10–20 s	[150,151]
Kroll’s reagent (10 mL of HNO_3_, 5 mL of HF, 85 mL of H_2_O)	Room temperature, 5–15 s	[82]
NaOH, 0.83 mol/L	Room temperature	[70]
Weck’s reagent 4 g of KMnO_4_, 1 g of NaOH, and 100 mL of distilled water for colour etching	Room temperature, 15–30 s	[152,153]
Barker’s reagent (5 mL HBF_4_ (40%) in 200 mL of distilled water)	Electrolytic etching, 20–30 V, direct current, 30 s, room temperature	[154,155]

**Table 4:** Characteristics of chemical extraction methods for deep etching and extraction of particles in aluminium alloys.

Chemical Extraction Method	Alloy	Characteristics	Reference
250 mL methyl alcohol solution with 2 wt.% KI, electrolytic etching, 2–5 V, 0.1–0.3 A	Al_6_Mn (14.3 at.% Mn) and Al_17_Mn (5.6 at.% Mn)	Preservation of quasicrystalline particles	[185]
Slightly acidic medium	Al-5,3 at.% Mn	Preservation of Al_6_Mn and Al_4_Mn, but dissolved IQC	[170]
perchloric acid (HClO_4_) and 20% methanol, electropolishing at −20 °C	Al_6_Mn (14.3 at.% Mn) and Al_17_Mn (5.6 at.% Mn)	Complete dissolution of IQC	[185]
NaOH aqueous solution (undefined composition)	Al–6Mn–2.5Be	Preservation of primary IQC-particles	[234]
5% aqueous solution of NaOH for 30 s	Al-Mn-Be-Cu alloy	Dissolution of IQC	[201]
electro-etched in a 250 mL methyl alcohol solution with 2 wt.% % KI; 10 V, 25 °C, 0.1 A/cm^2^	Melt-spun Al-Mn	3D morphology of IQC was preserved	[226]
10% nitric acid in ethanol for 5–10 min. They could dissolve the magnesium-rich matrix to	AZ magnesium alloy	Preservation of Al-Mn-based decagonal quasicrystals and some other crystalline phases	[235]
5% nitric acid solution in 95% ethanol	Mg-Al-Zn alloys	Preservation of IQC	[167]
2.5 mL HCl, 5 mL HNO_3,_ and 1 mL HF	Al-Mn-Be and Al-Mn-Be-Cu alloys	Strong attack on the intermetallic phases	[236]
5 mL HNO_3_, 2,5 mL HCl, and 70 mL alcohol for 30 s	Al-Mn-Be and Al-Mn-Be-Cu alloys	Complete dissolution of IQC	[236]
10 g of iodine and 25 g of tartaric acid in 250 mL of methanol	Al-Ti-B	Preservation of TiB_2_ and Al_3_Ti	[230]
Variable contents of iodine and tartaric acid in methanol	Al-Mn-Be and Al-Mn-Be-Cu alloys	Preservation of IQC and other intermetallic phases	[237]

## Data Availability

No new data were created or analyzed in this study. Data sharing is not applicable to this article.

## Abbreviations

QA	Quasicrystalline Approximant
EELS	Electron Energy Loss Spectroscopy
HAADF	High-Angle Annular Dark Field
TEM	Transmission Electron Microscopy
IQC	Icosahedral Quasicrystal
DQC	Decagonal Quasicrystal
STEM	Scanning TEM
EDS	Energy-Dispersive X-Ray Spectroscopy
WDS	Wavelength Dispersive X-Ray Spectroscopy
LM	Light Microscopy
SEM	Scanning Electron Microscopy
FIB	Focus Ion Beam
EBSD	Electron-BackScattered Diffraction
t-EBSD	transmission EBSD
HR SEM	High-Resolution SEM
XRD	X-ray Diffraction
APT	Atom-Probe Tomography
TKD	transmission Kikuchi diffraction
RE	rare-earth metal
